# Supplementary figures and images for: Evolutionary repair: Changes in multiple functional modules allow meiotic cohesin to support mitosis
Source: PLoS Biol. 2020 Mar 10;18(3):e3000635. doi: 10.1371/journal.pbio.3000635 (PMC7138332; doi:10.1371/journal.pbio.3000635)

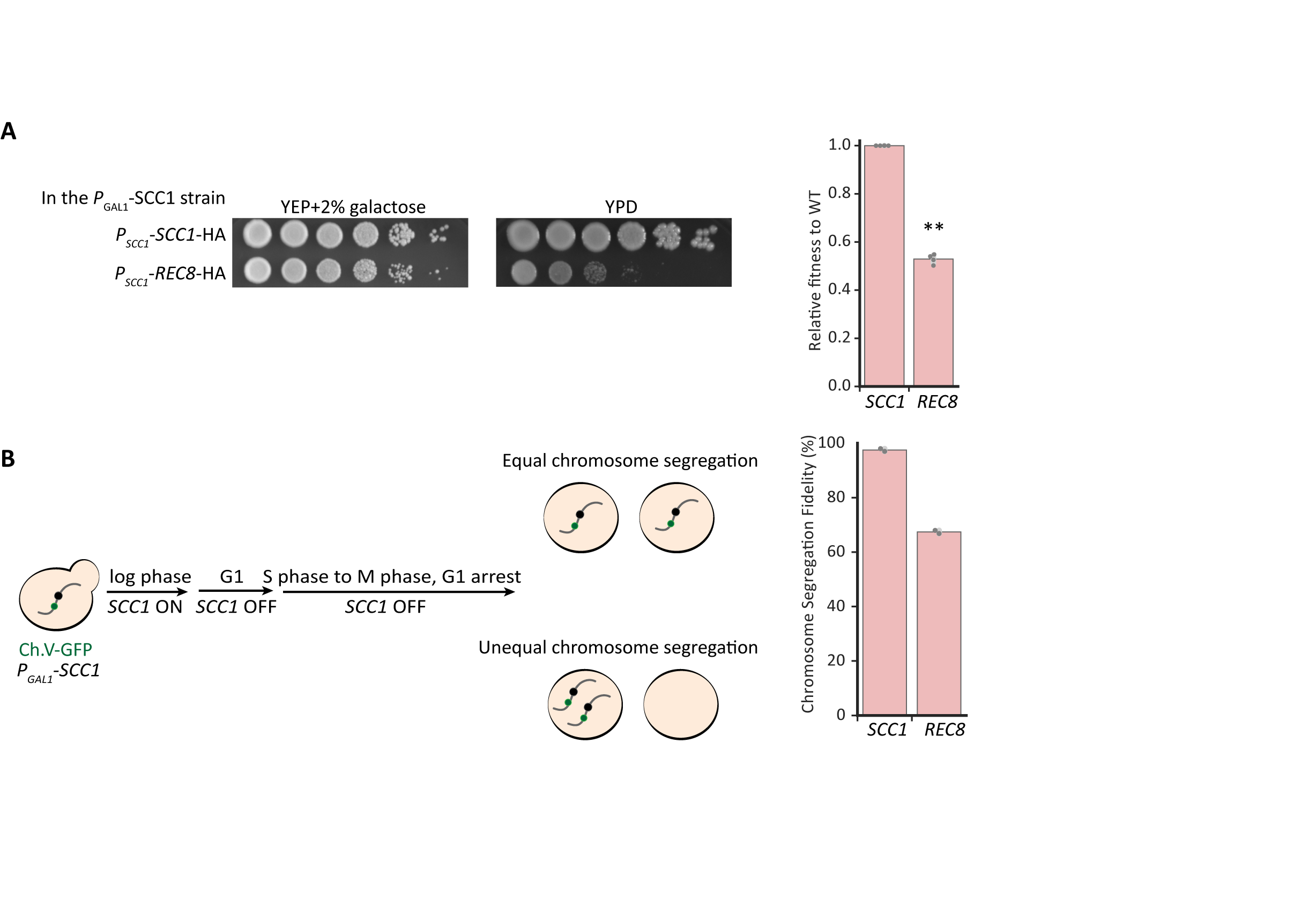

Supplement: S1 Fig — We used a PGAL1-SCC1 PSCC1-REC8 strain to examine the effect of acutely expressing Rec8 as the sole kleisin. Cells were propagated in galactose-containing media, arrested in G1, and then released into glucose-containing media to repress Scc1. (A) The PSCC1-REC8 strain grows poorly when SCC1 expression is turned off. Left: Cells were grown in YEP containing 2% galactose to the same density and serially diluted on YEP containing 2% galactose or 2% glucose, in which the GAL1 promoter was repressed by glucose. Right: The fitness of a PGAL1-SCC1 PSCC1-REC8 strain relative to that of wild type in YPD. The darker gray points represent the values of three biological replicates, and the thinner gray bar represents one standard deviation on each side of the mean of these measurements (two-tailed Student t test, **p < 0.01). (B) The fidelity of chromosome segregation of the Rec8-expressing strain is 30% lower than that of wild type. PGAL1-SCC1 PSCC1-REC8 cells were grown in YEP containing 2% galactose to log phase, transferred to YEP containing 2% raffinose and α-factor to repress SCC1 expression and arrest them in G1, prior to release into YPD to resume cell cycle with SCC1 expression repressed. Once cells had entered S phase, α-factor was added again to prevent cells entering a second cell cycle. Chromosome segregation fidelity was measured as the fraction of G1-arrested cells in a population showing one GFP dot, representing one copy of Chromosome 5, after one mitotic cell division. At least 100 cells were imaged in each experiment. The darker gray points represent the values of two biological replicates, and the thinner gray bar represents one standard deviation on each side of the mean of these measurements. Data associated with S1A and S1B Fig can be found in S1 Data. GAL1, galactose metabolism 1; GFP, green fluorescent protein; Rec8, recombination 8; Scc1, sister chromosome cohesion 1; YEP, yeast extract and peptone; YPD, yeast extract, peptone, and dextrose. (TIF) [file pbio.3000635.s001.tif]

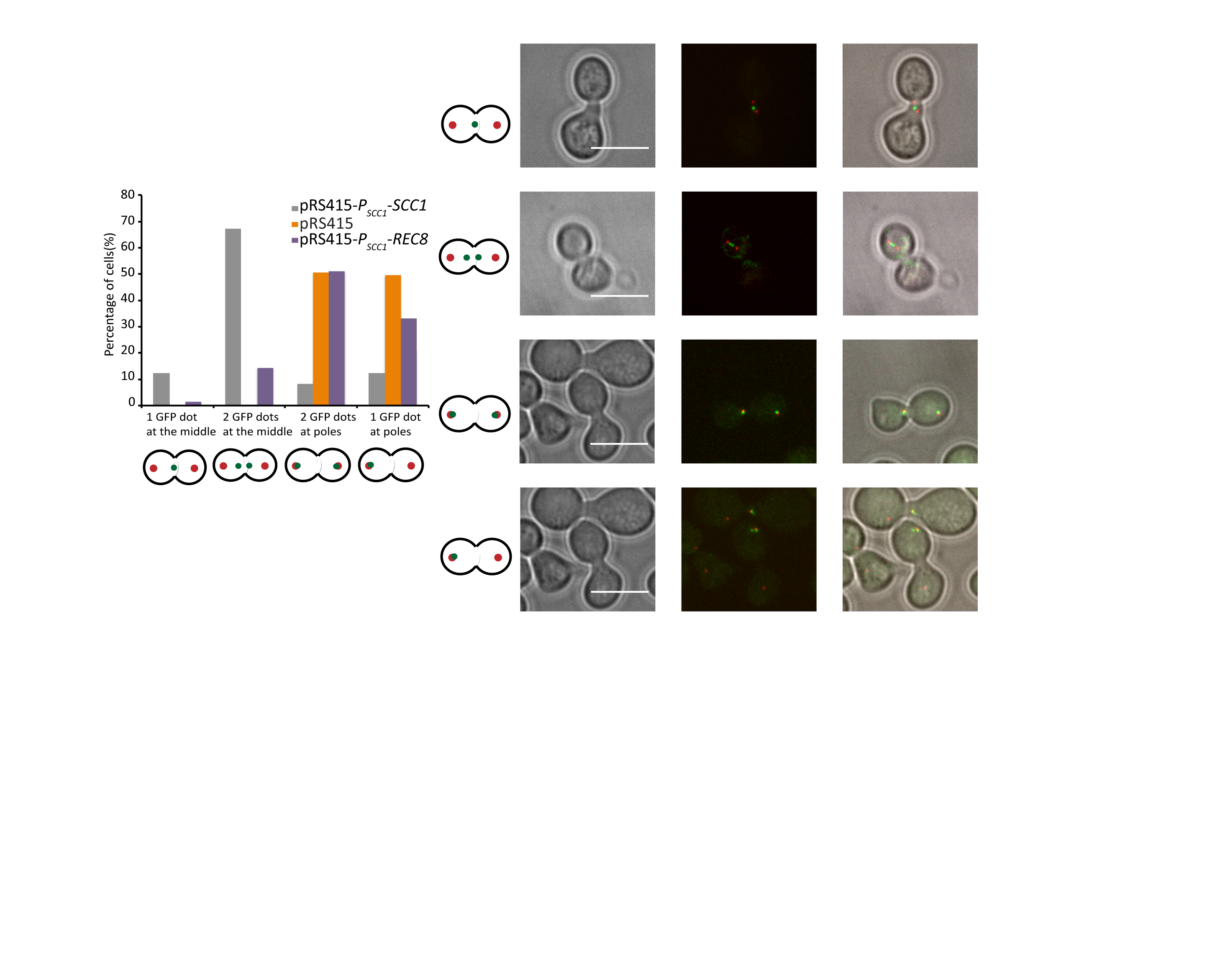

Supplement: S2 Fig — The yeast strain PMET-CDC20-3xHA PGAL1-SCC1-3xHA CEN15::LacO PCUP1-GFP-LacI SPC42-mCherry was transformed with a pRS415-based plasmid of PSCC1- SCC1, PSCC1-REC8, or an empty plasmid. Cells were cultured in CSM-Met-Leu containing galactose to log phase and switched to CSM-Met-Leu containing raffinose and α-factor to be synchronized in G1. Then, to repress the SCC1 expression and arrest cells in metaphase, cells were released into YEP containing glucose and methionine for one cell cycle. The centromere of Chromosome 15 was marked by GFP and spindle pole bodies were labeled by SPC42-mCherry. Cells showing one or two GFP dots in the middle of two spindle pole bodies represent bi-oriented sister kinetochores under tension exerted by the spindle. The lack of sister chromosome cohesion leads to sister chromosomes of Chromosome 15 separating in prometaphase, resulting either in one GFP dot at each spindle pole body or two GFP dots at one of the spindle pole bodies. At least 100 cells were imaged and analyzed in each population. Illustrative microscopy images are shown in the right; the scale bar is 10 μm. Data associated with this figure can be found in S1 Data. CSM, complete synthetic media; GFP, green fluorescent protein; SCC1, sister chromosome cohesion 1; YEP, yeast extract and peptone. (TIF) [file pbio.3000635.s002.tif]

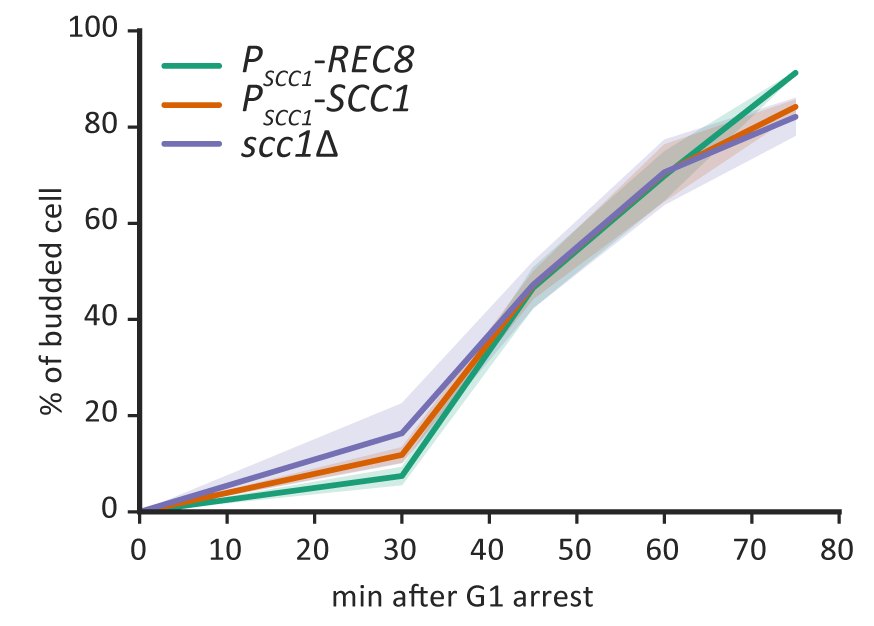

Supplement: S3 Fig — Cells were arrested in G1 and then released as described in Fig 1C. The y-axis shows the fraction of budded cells in a population, measured as the budding index. At least 100 cells were examined at each time point for each experiment. The mean (solid line) and standard deviation (shaded region) of three biological replicates for each population are shown. Data associated with this figure can be found in S1 Data. Rec8, recombination 8 (TIF) [file pbio.3000635.s003.tif]

**A**

Chr. 1

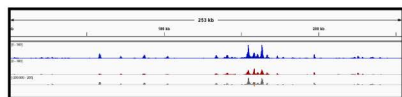

Chr. 2

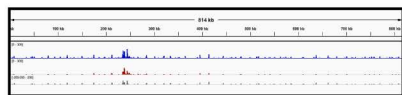

Chr. 3

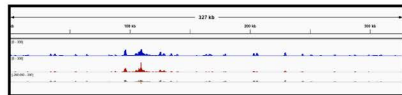

Chr. 4

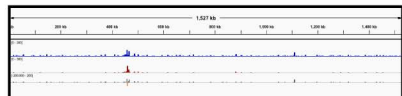

Chr. 5

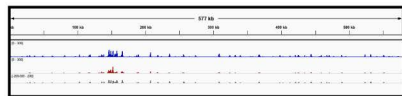

Chr. 6

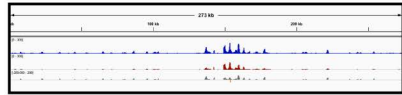

Chr. 7

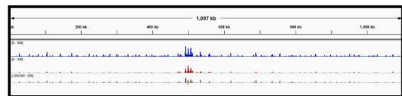

Chr. 8

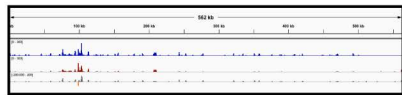

Chr. 9

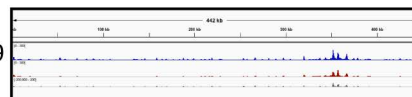

Chr. 10

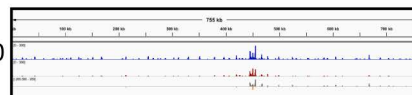

Chr. 11

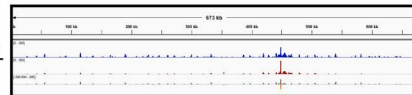

Chr. 12

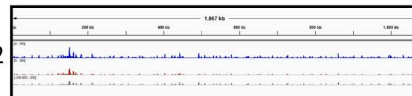

Chr. 13

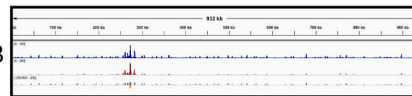

Chr. 14

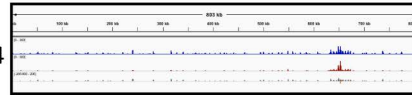

Chr. 15

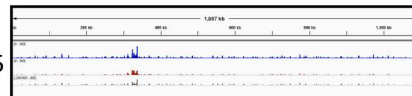

Chr. 16

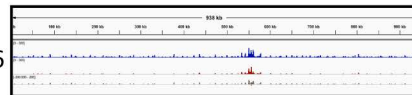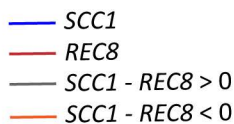

**B**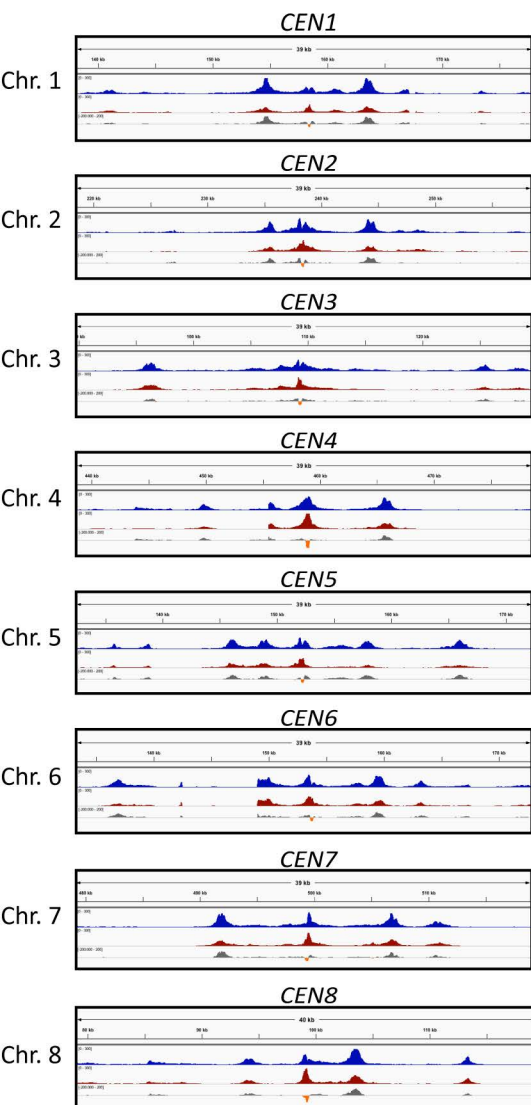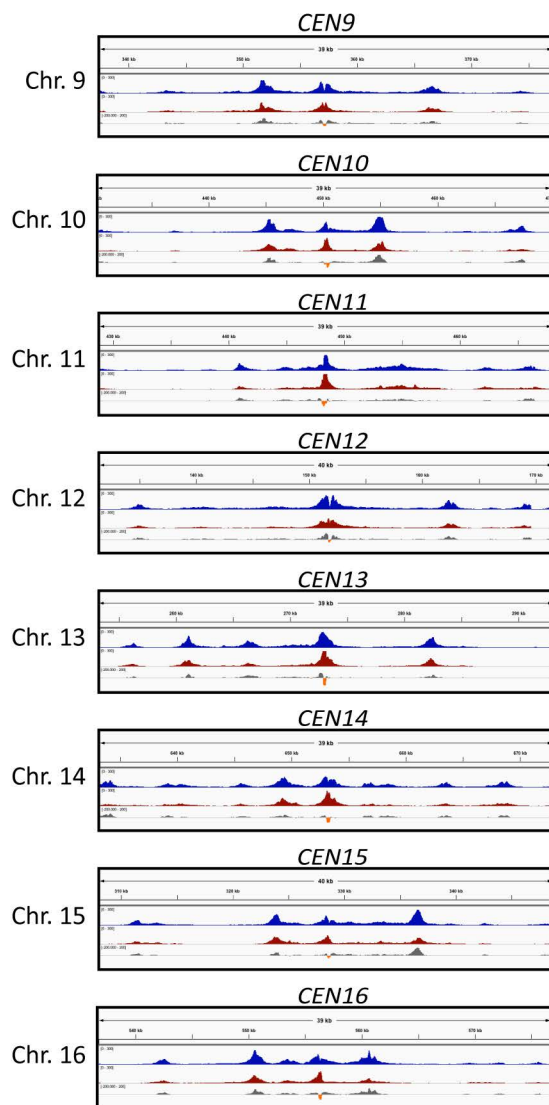

— *SCC1*  
 — *REC8*  
 — *SCC1 - REC8 > 0*  
 — *SCC1 - REC8 < 0*

Supplement: S4 Fig — The ChIP-Seq data for all sixteen chromosomes (Chromosomes 3 and 15 are also shown in Fig 2D). Read depths calibrated to an internal control of the S. pombe genome are shown on the y-axis as reads per million (RPM, 0–300). The enrichment of Scc1 and Rec8 is shown in blue and red, respectively. The difference in the read depth between Scc1 and Rec8 is shown in the last track of each panel, in gray where Scc1’s signal is higher than Rec8’s, and in orange where Rec8’s signal is higher than Scc1’s. (A) ChIP-Seq data of individual chromosomes. (B) ChIP-Seq data of individual centromeres extending 20 kb on either side of the centromeres. Graphs were prepared using the Integrated Genomic Viewer [33]. ChIP-Seq, chromatin immunoprecipitation sequencing; Rec8, recombination 8; Scc1, sister chromosome cohesion 1. (PDF) [file pbio.3000635.s004.pdf]

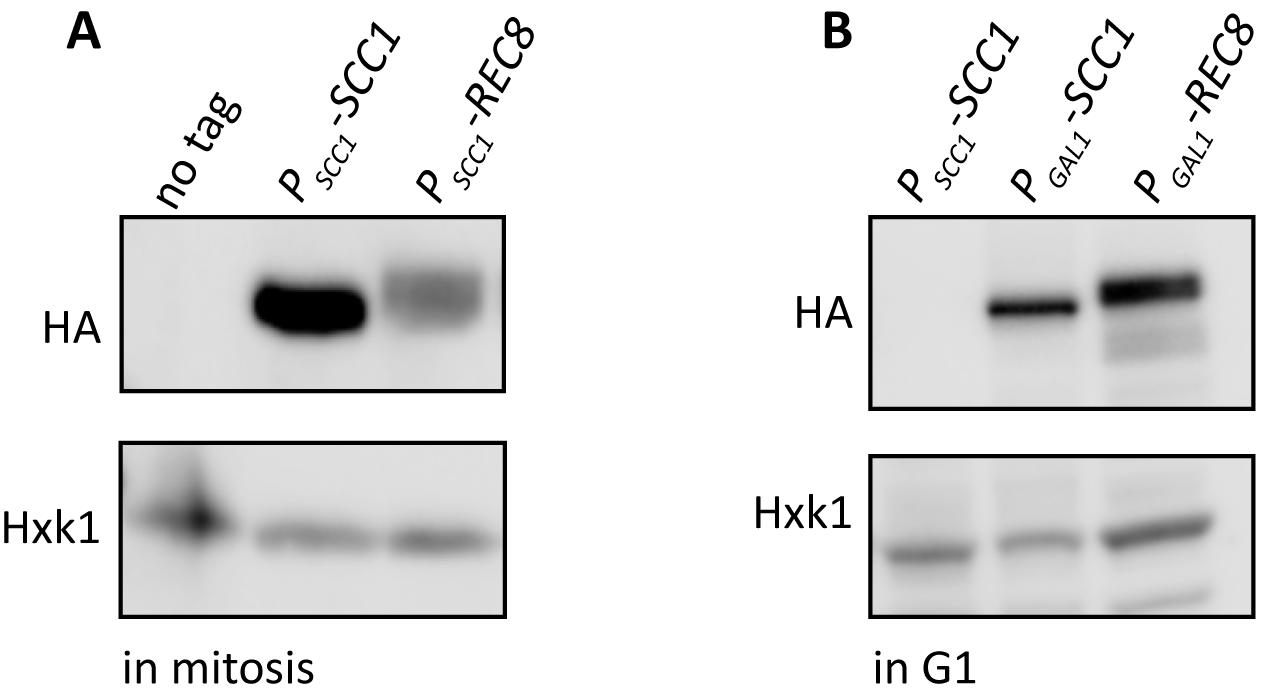

Supplement: S5 Fig — (A) Protein levels of two kleisins in mitosis. Cells were processed as described in Fig 2C and cell extracts were obtained by alkaline lysis prior to analysis by western blotting. Kleisin proteins were detected by anti-HA antibody and Hxk1 was used as a loading control. (B) Protein levels of two ectopically expressed kleisins in G1. Cells were processed as described in Fig 2E and cell extracts were obtained by alkaline lysis prior to analysis by western blotting. The PSCC1-SCC1-HA strain was used as a negative control because the endogenous SCC1 gene is not expressed in G1. Hxk1 was used as a loading control. Raw images associated with S5A and S5B Fig can be found in S1 Raw Image. ChIP, chromatin immunoprecipitation; HA, hemagglutinin; Hxk1, hexokinase; Rec8, recombination 8; Scc1, sister chromosome cohesion 1. (TIF) [file pbio.3000635.s005.tif]

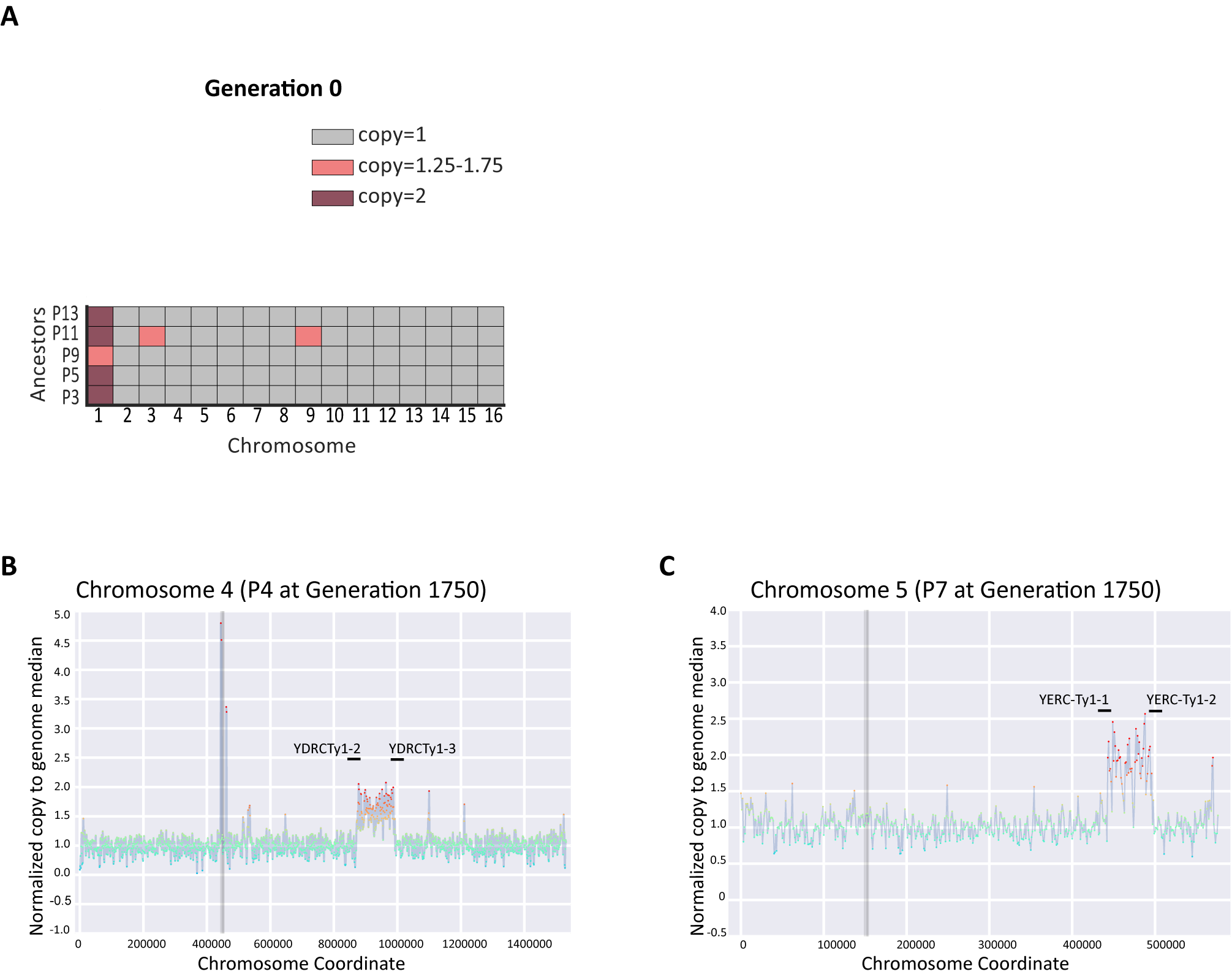

Supplement: S6 Fig — (A) Chromosomal copy number of five Rec8-expressing ancestors. The copy number of each chromosome was calculated by normalizing the median read depth of each chromosome to the median read depth over the entire genome. Gray marks one copy, dark red marks two copies, and pink marks 1.25–1.75 copies, suggesting that part of the population was disomic. Data associated with this figure can be found in S1 Data. (B) The copy number data of Chromosome 4 of population P4 at generation 1,750. (C) The copy number data of Chromosome 5 of population P7 at generation 1,750. In (B) and (C), copy numbers normalized to the median read depth of each sequenced genome are shown. Chromosomal regions showing copy number below 1.5 are marked with green, and regions showing copy number equal to 2 are marked with red. The position of centromeres is marked with a vertical gray line. The transposons on the edges of duplicated regions are annotated with horizontal black bars. Rec8, recombination 8. (TIF) [file pbio.3000635.s006.tif]

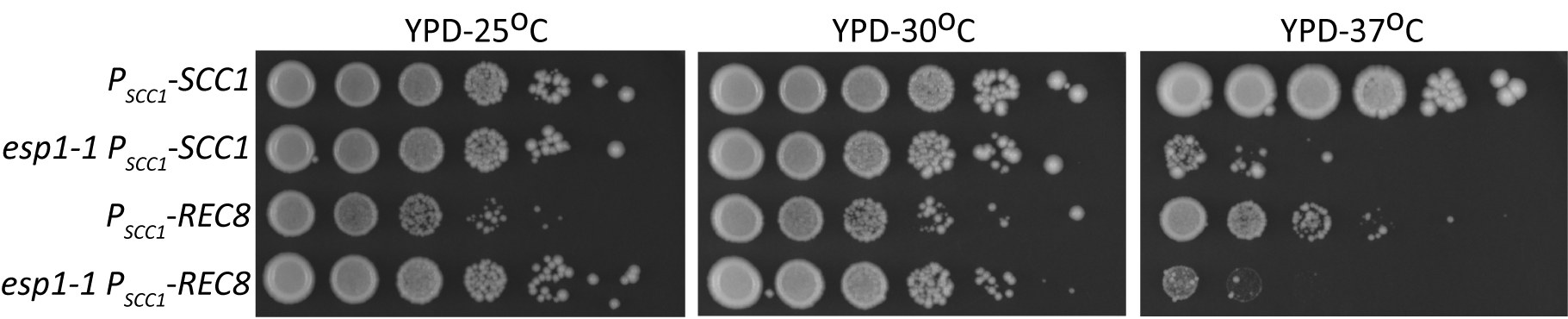

Supplement: S7 Fig — esp1-1, the known temperature-sensitive mutation that inactivates separase activity [32], was introduced to the Rec8-expressing strain by sporulating a heterozygous diploid strain (PSCC1-SCC1/PSCC1-REC8 ESP1/esp1-1). Haploid progeny carrying four different genotypes (SCC1, SCC1 esp1-1, REC8, and REC8 esp1-1) were selected. These four strains were subjected to serial dilutions and spotted on YPD. Their growth was measured at 25°C, 30°C, and 37°C. esp1-1, extra spindle pole bodies 1–1; Rec8, recombination 8; SCC1, sister chromosome cohesion 1; YPD, yeast extract, peptone, and dextrose. (TIF) [file pbio.3000635.s007.tif]

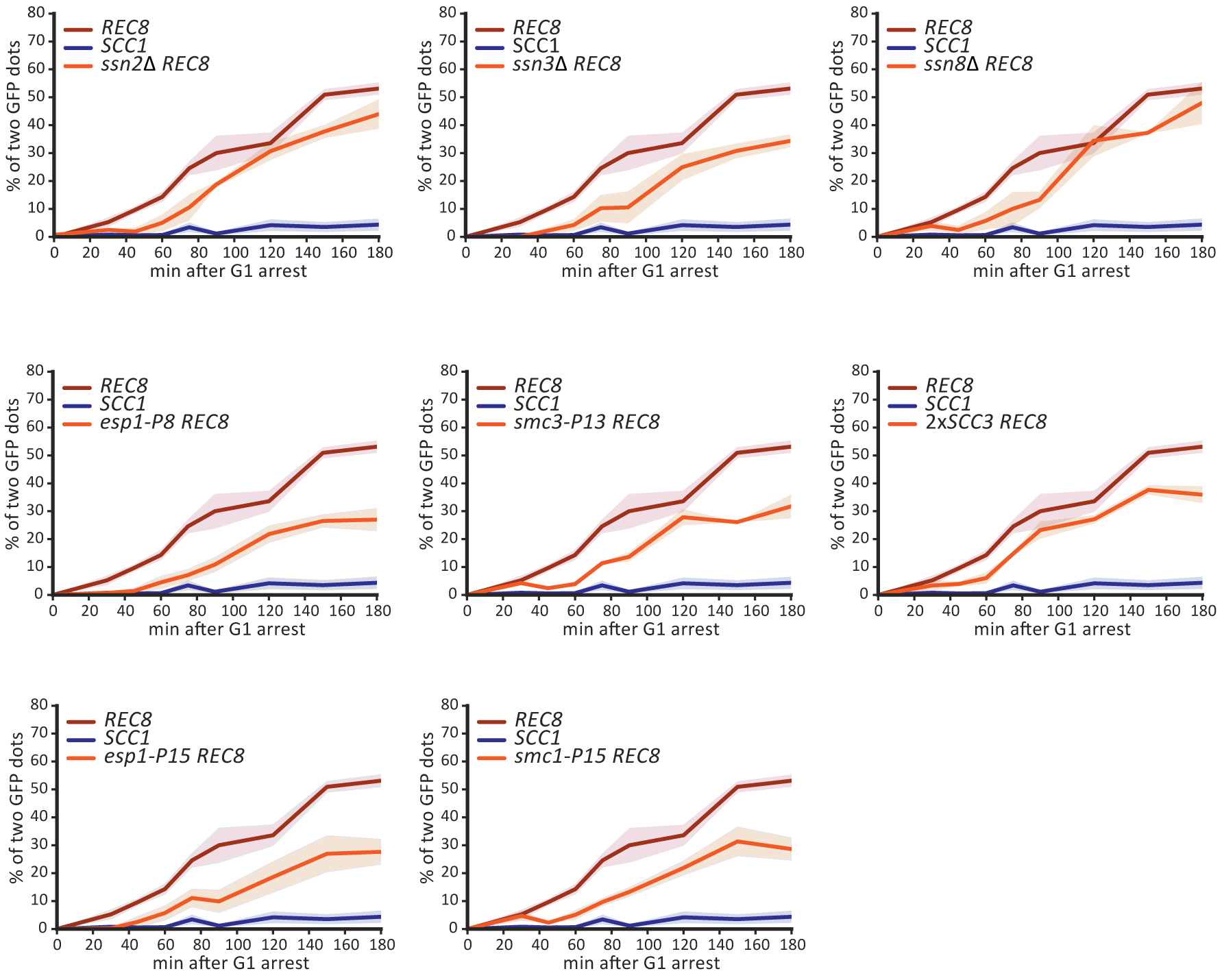

Supplement: S8 Fig — The time courses of sister chromosome separation for the experiment shown in Fig 5A, which presents the data at 150 minutes after release from a G1 arrest. At least 100 cells were imaged at each time point for each experiment. The mean and standard deviation of three biological replicates are shown in the solid line and shading, respectively. Data associated with this figure can be found in S1 Data. (TIF) [file pbio.3000635.s008.tif]

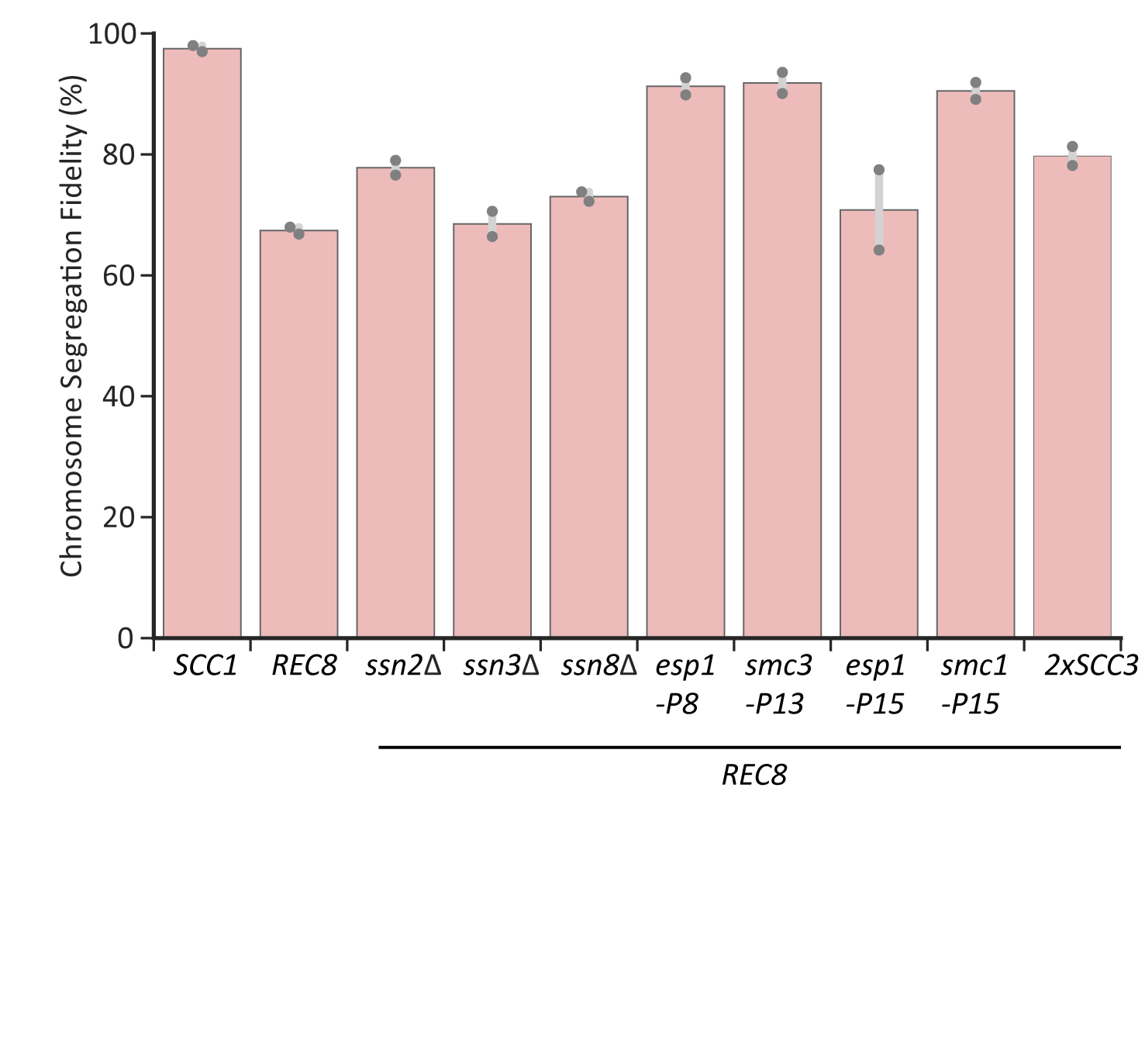

Supplement: S9 Fig — Cells were prepared as in S1B Fig to examine the fidelity of chromosome segregation in a single mitotic cell division. Gene deletion for three components of the Cdk8 complex was used to approximate the effect of the mutations of these genes found in evolved populations. At least 100 cells were imaged in each experiment. The darker gray points represent the values of two biological replicates and the thinner gray bar represents one standard deviation on each side of the mean of these measurements. Data associated with this figure can be found in S1 Data. Cdk8, cyclin dependent kinase 8; Rec8, recombination 8. (TIF) [file pbio.3000635.s009.tif]

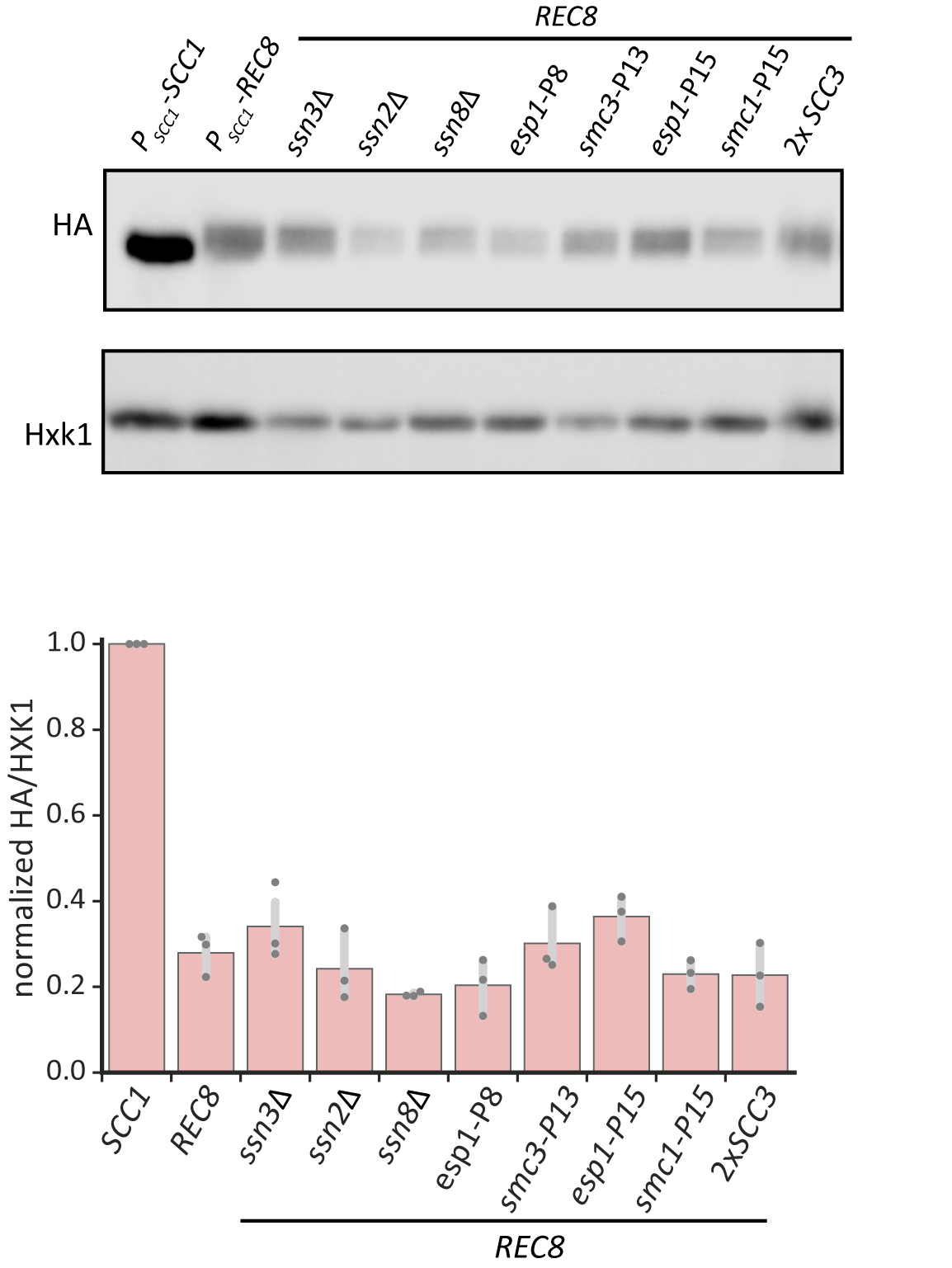

Supplement: S10 Fig — Strains with individual reconstructed mutations were synchronized in G1, released into cell cycle, and then arrested in mitosis in YPD containing benomyl. Protein samples were collected by alkaline lysis and analyzed by western blotting. Both Scc1 and Rec8 were tagged with 3xHA at their C termini, and anti-HA antibody was used for their detection. Hxk1 was used as loading control. In the bar graph, the darker gray points represent the values of three biological replicates, and the thinner gray bar represents one standard deviation on each side of the mean of these measurements. Raw images and data associated with this figure can be found in S1 Raw Image and S1 Data, respectively. Hxk1, hexokinase; Rec8, recombination 8; Scc1, sister chromosome cohesion 1; YPD, yeast extract, peptone, and dextrose; 3xHA, 3 copies of hemagglutinin. (TIF) [file pbio.3000635.s010.tif]

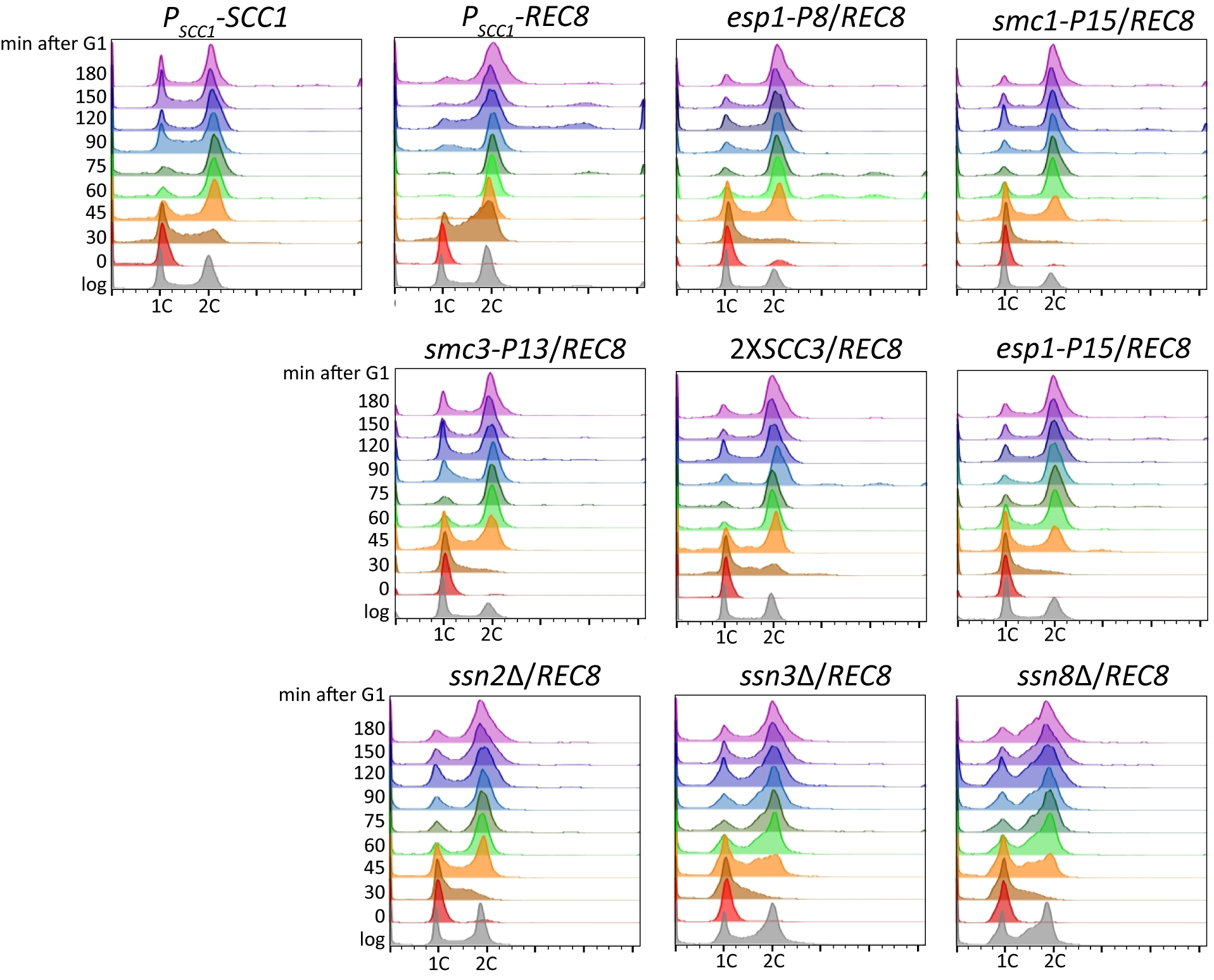

Supplement: S11 Fig — The full-time course data for the experiments summarized in Fig 6A, which shows the data from 0 to 75 minutes after release from a G1 arrest. Individual cohesin-related mutations, deletion of genes encoding the Cdk8 complex, and two integrated copies of SCC3 were engineered separately into the PSCC1-REC8 PGAL1-SCC1 background. Cells were allowed to proceed through a synchronous cell cycle as in Fig 1D and were collected for fixation every 15 or 30 minutes following release from a G1 arrest to analyze their DNA content. Cdk8, cyclin dependent kinase 8; Rec8, recombination 8; SCC3, sister chromosome cohesion 3. (TIF) [file pbio.3000635.s011.tif]

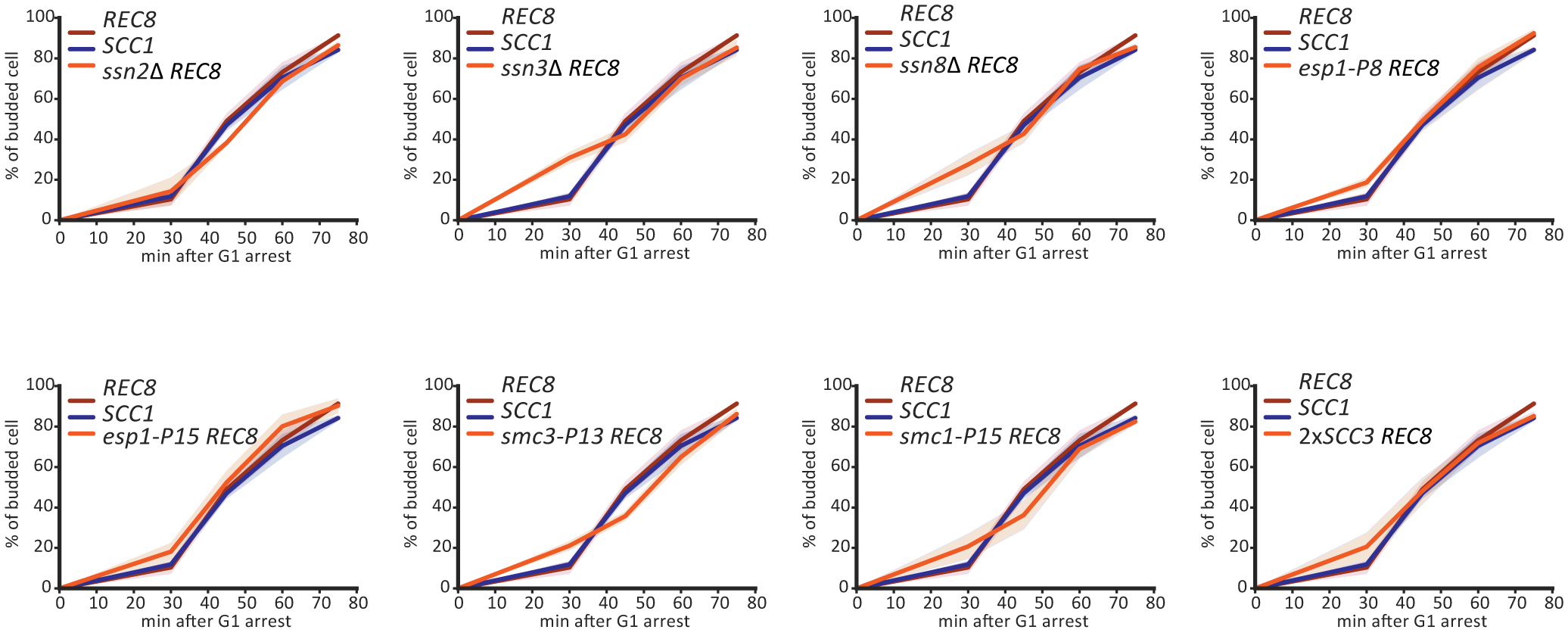

Supplement: S12 Fig — Cells were arrested in G1 and then released as described in Fig 6A. The y-axis shows the fraction of budded cells in a population, measured as the budding index. Strains carrying single reconstructed mutations (ssn2Δ, ssn3Δ, ssn8Δ, esp1-P8, esp1-P15, smc3-P13, smc1-P15, or two copies of SCC3) are compared with the wild type and the Rec8-expressing strain. The mean (solid line) and standard deviation (shaded region) of three biological replicates for each strain are shown. Data associated with this figure can be found in S1 Data. Rec8, recombination 8; SCC3, sister chromosome cohesion 3. (TIF) [file pbio.3000635.s012.tif]

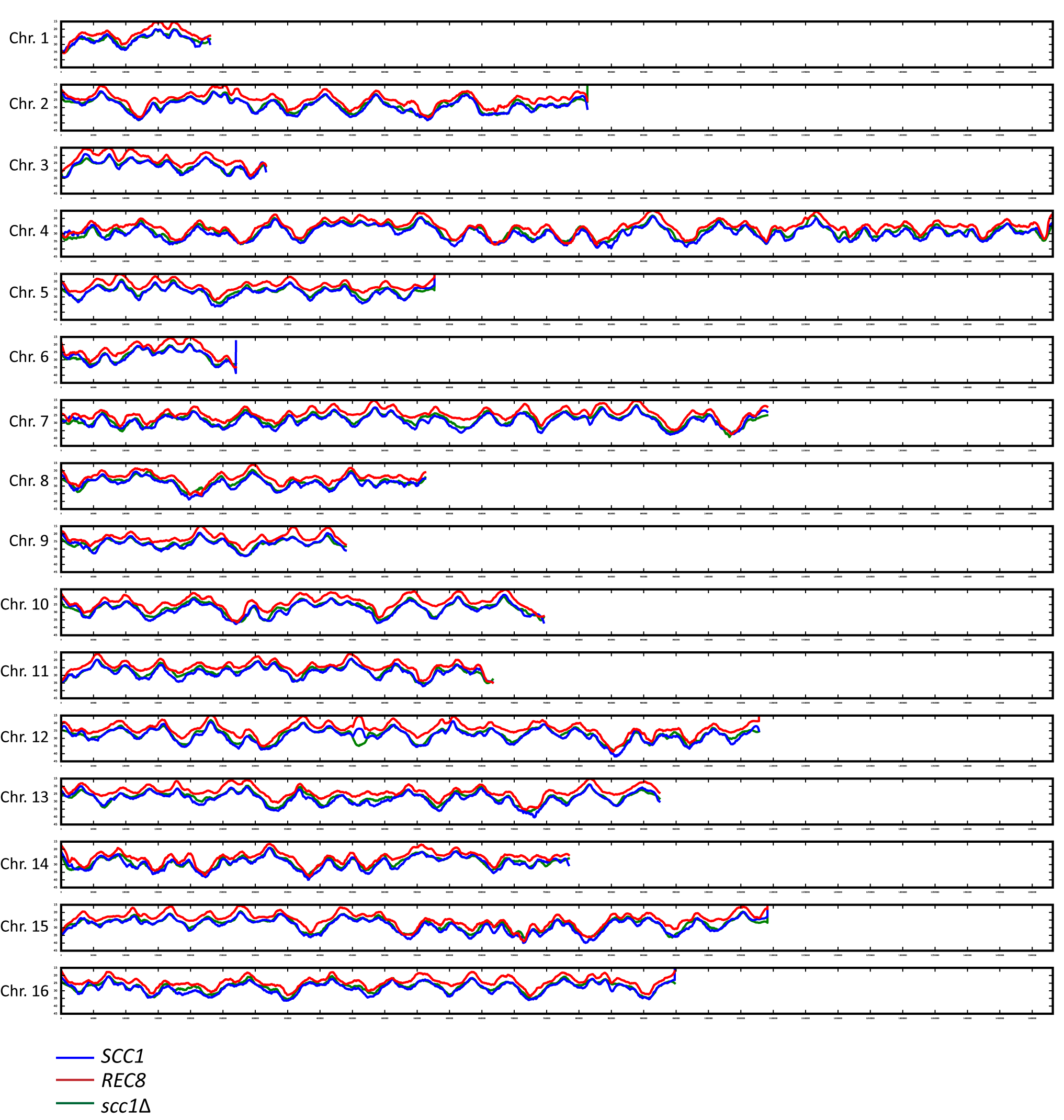

Supplement: S13 Fig — The mean replication profile of two experiments is shown. The replication profile of each strain is color-coded (SCC1 in blue, REC8 in red, and scc1Δ in green) and arranged by order of chromosome. The y-axis represents Trep, the time at which 50% of cells in a population completes replication at a given genomic locus (See Materials and methods for detailed analysis). Rec8, recombination 8; SCC1, sister chromosome cohesion 1. (TIF) [file pbio.3000635.s013.tif]

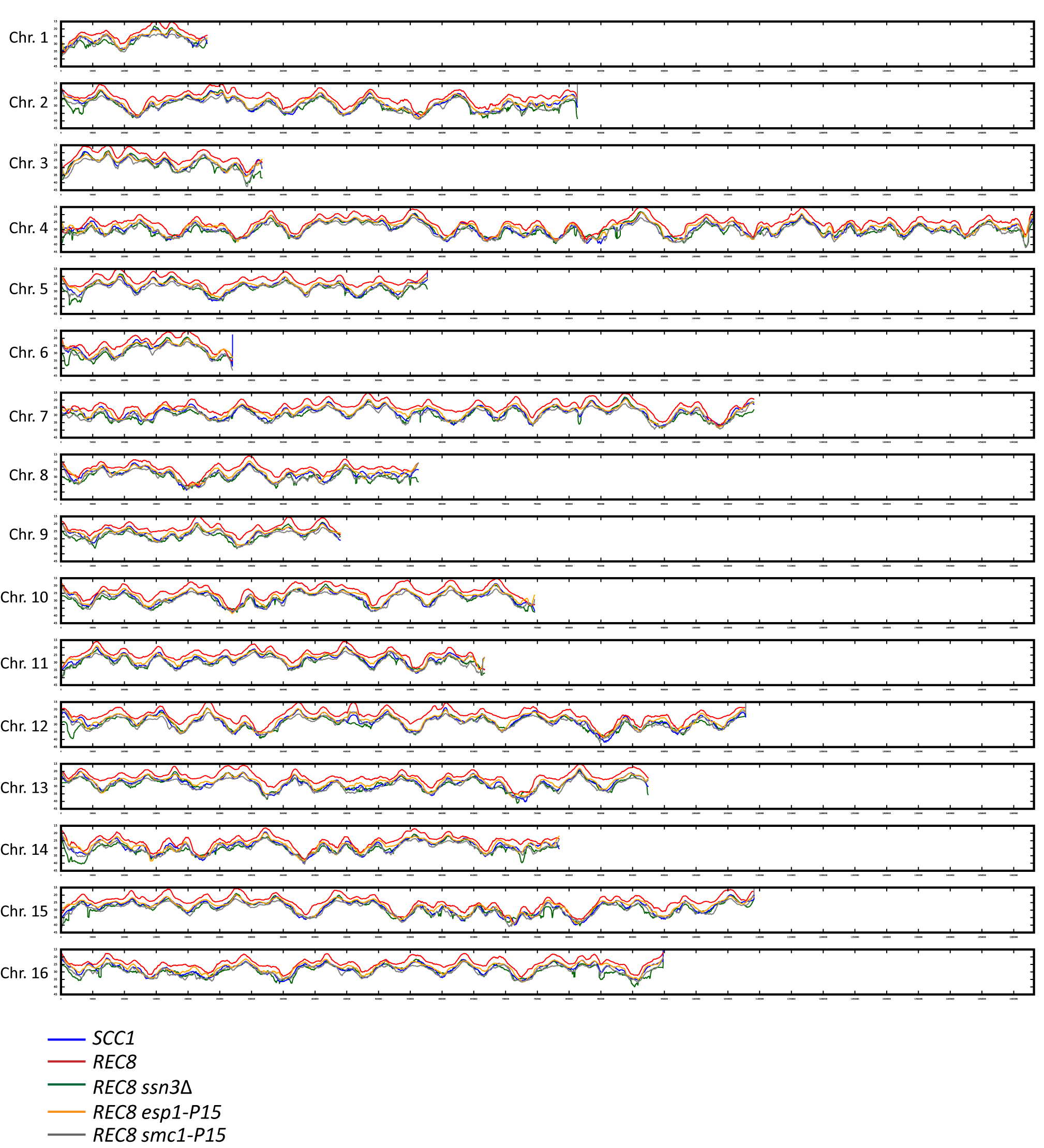

Supplement: S14 Fig — These replication profiles are the data shown in Fig 6E. The mean replication profile from two experiments is color-coded by strain and arranged by order of chromosome. The y-axis represents Trep, the time at which 50% of cells in a population completes replication at a given genomic locus (See Materials and methods for detailed analysis). Rec8, recombination 8. (TIF) [file pbio.3000635.s014.tif]

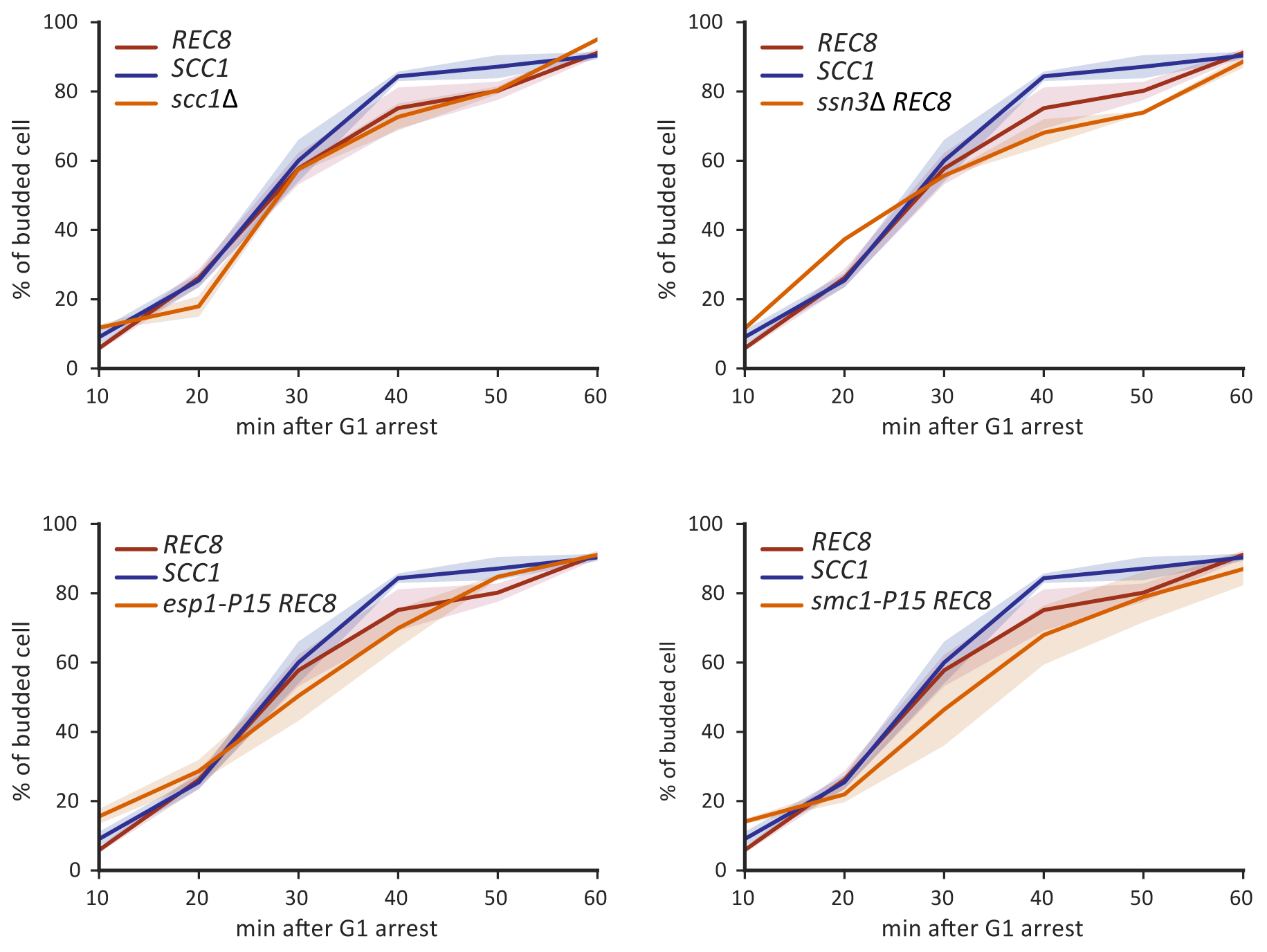

Supplement: S15 Fig — Cells were arrested in G1 and then released as described in Figs 6E, S13 and S14. The y-axis shows the fraction of budded cells in a population, measured as budding index. The mean (solid line) and standard deviation (shaded region) of two biological replicates for each strain are shown. Data associated with this figure can be found in S1 Data. (TIF) [file pbio.3000635.s015.tif]

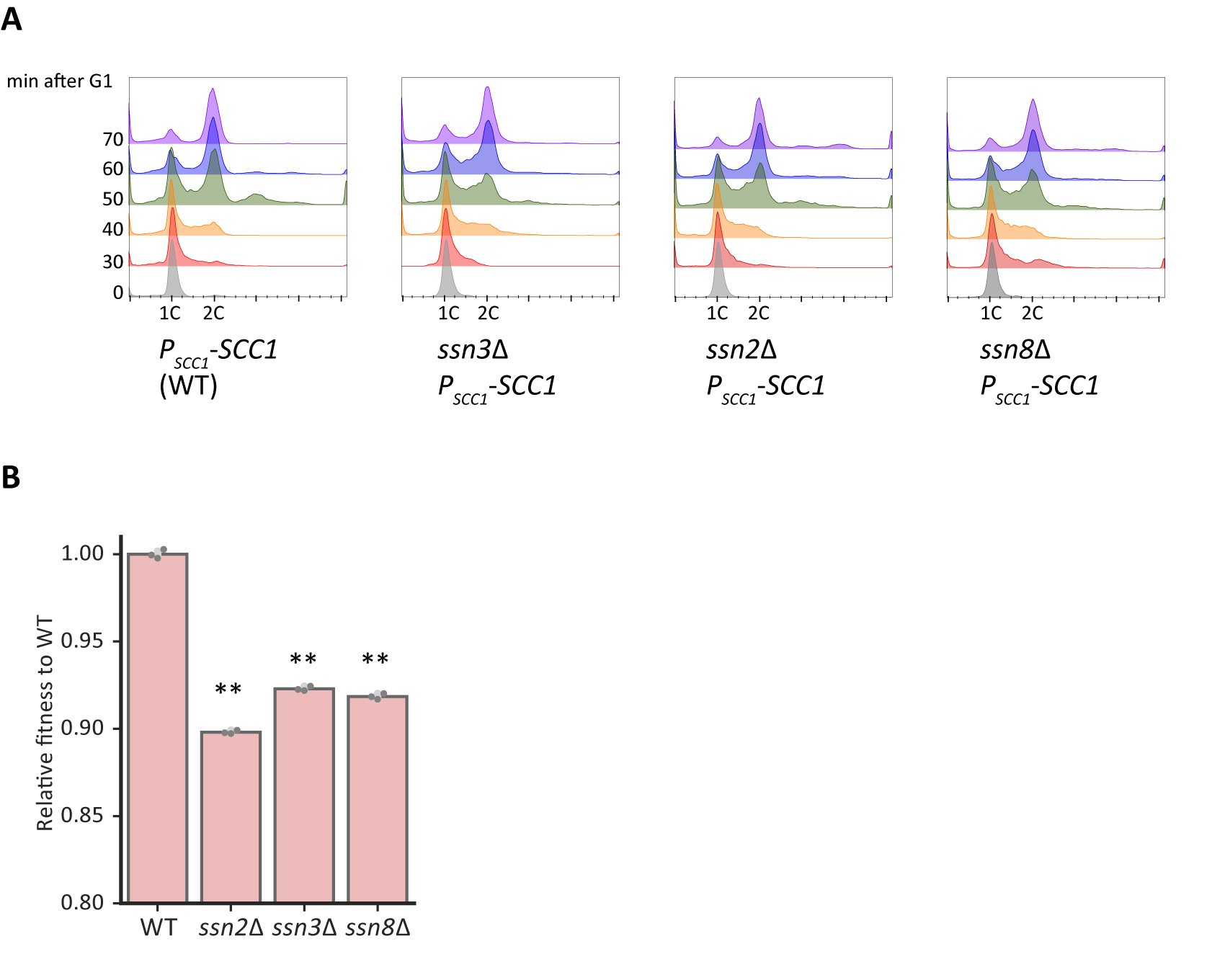

Supplement: S16 Fig — (A) The cell cycle progression profiles of ssn2Δ, ssn3Δ, or ssn8Δ strains compared to a wild-type control after release from a G1 arrest. (B) The fitness of ssn2Δ, ssn3Δ, or ssn8Δ strains relative to wild type, measured by competitive fitness assay. The darker gray points represent the values of three biological replicates and the thinner gray bar represents one standard deviation on each side of the mean of these measurements. The statistical significance between data from wild type and each mutant strain was calculated by two-tailed Student t test, **p < 0.01. Data associated with this figure can be found in S1 Data. Cdk8, cyclin dependent kinase 8. (TIF) [file pbio.3000635.s016.tif]

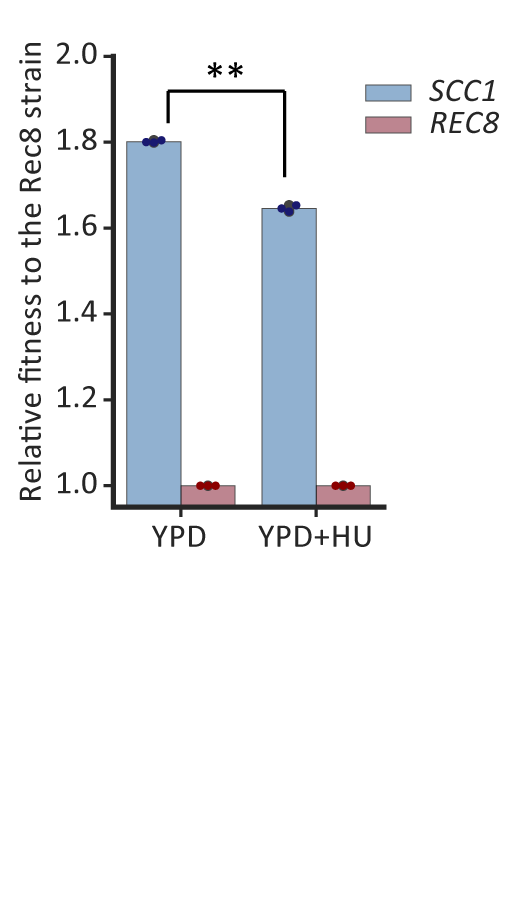

Supplement: S17 Fig — The fitness of wild-type strains relative to that of the Rec8-expressing strain were measured in YPD and YPD containing 12.5 mM HU. The colored points represent the values of three biological replicates, and the darker gray bar represents one standard deviation on each side of the mean of these measurements (two-tailed Student t test, **p < 0.01). Data associated with this figure can be found in S1 Data. HU, hydroxyurea; Rec8, recombination 8; YPD, yeast extract, peptone, and dextrose. (TIF) [file pbio.3000635.s017.tif]
