# Supplementary material for: Evolutionary repair: Changes in multiple functional modules allow meiotic cohesin to support mitosis
Source: PLoS Biol. 2020 Mar 10;18(3):e3000635. doi: 10.1371/journal.pbio.3000635 (PMC7138332; doi:10.1371/journal.pbio.3000635)
Supplement: S2 Table — (PDF) [file pbio.3000635.s019.pdf]

S2 Table. Adaptive mutations fixed in each evolved population at generation 1750

Population	Gene	Nucleotide Change	Amino Acid change
**P1**	*SSN2*	3031 C -> T	1011 stop codon
	ESP1	2537 C -> T	846 C -> Y
**P2**	*SSN2*	2774 T -> G	925 I -> S
	SMC3	895 C -> G	299 A -> P
**P3**	*SSN8*	817 +T	273 stop codon
	SWI4	1906 T -> A, 1993 C -> A	635 N -> Y, 665 G -> C
	CLN2	745 G -> A	249 stop codon
**P4**	*SSN3*	108 del	36 stop codon
	SMC3	1131 T -> G	377 Q -> H
**P5**	*SSN2*	2555 T -> A	852 stop codon
	SMC3	88 T -> C	30 I -> V
**P6**	*SRB7*	230 T -> G	77 I -> S
	ESP1	3580 G -> C	1194 H -> D
**P7**	*SSN2*	3258 C -> A	1086 stop codon
	SWI6	2144 C -> A	715 stop codon
**P8**	*ESP1*	2537 C -> T	846 C -> Y
	SMC3	1622 C -> T	941 G -> D
**P9**	*SSN3*	253 del	94 stop codon
	CLN2	622 C -> T	208 E -> K
**P10**	*SRB8*	4239 C -> G	1413 N -> K
**P11**	*NUT2*	134 C -> A	45 A -> D
	SMC3	2530 G -> C	844 L -> V
	SMC1	2232 G -> T	744 E -> D
	MBP1	1512 A -> +T, 1513 C -> T	510 stop codon
**P12**	*SIN4*	2838 del	947 T -> L
	SRB8	1058 del	354 stop codon
	SMC3	2351 G -> T	784 F -> K
**P13**	*SRB8*	3095 C -> A	1032 stop codon
	SMC3	3623 G -> A	1208 T -> M
	CLN2	375 C -> T	125 stop codon
**P14**	*SSN8*	197 A -> T	66 stop codon
**P15**	*SSN2*	3058 T -> A	1020 stop codon
	SMC1	1253 A -> C	418 Q -> P
	ESP1	3719 A -> T	1240 I -> N
	MBP1	1268 C -> A	423 stop codon
