# Supplementary material for: Evolutionary repair: Changes in multiple functional modules allow meiotic cohesin to support mitosis
Source: PLoS Biol. 2020 Mar 10;18(3):e3000635. doi: 10.1371/journal.pbio.3000635 (PMC7138332; doi:10.1371/journal.pbio.3000635)
Supplement: S3 Table — ChIP, chromatin immunoprecipitation; qPCR, quantitative polymerase chain reaction. (PDF) [file pbio.3000635.s020.pdf]

S3 Table. Primers used for ChIP-qPCR

Primer	Chromosomal Position	Sequence
AM792	IV-c1	ACACGAGCCAGAAATAGTAAC
AM793	IV-c1	TGATTATAAGCATGTGACCTTT
AM794	IV-c2	CCGAGGCTTTCATAGCTTA
AM795	IV-c2	ACCGGAAGGAAGAATAAGAA
AM1319	IV-p1	ATGATTCAATGGATTTAGCC
AM1320	IV-p1	GTCAGTCTTATGCTGTTCCC
AM782	IV-a1	AGATGAAACTCAGGCTACCA
AM783	IV-a1	TGCAACATCGTTAGTTCTTG
oPH622	Spombe-otr1	AATTGTGGTGGTGTGGTAATAC
oPH623	Spombe-otr1	GGGTTCATCGTTTCCATTCAG
