# Supplementary material for: Evolutionary repair: Changes in multiple functional modules allow meiotic cohesin to support mitosis
Source: PLoS Biol. 2020 Mar 10;18(3):e3000635. doi: 10.1371/journal.pbio.3000635 (PMC7138332; doi:10.1371/journal.pbio.3000635)

Fig 2A. Raw Image

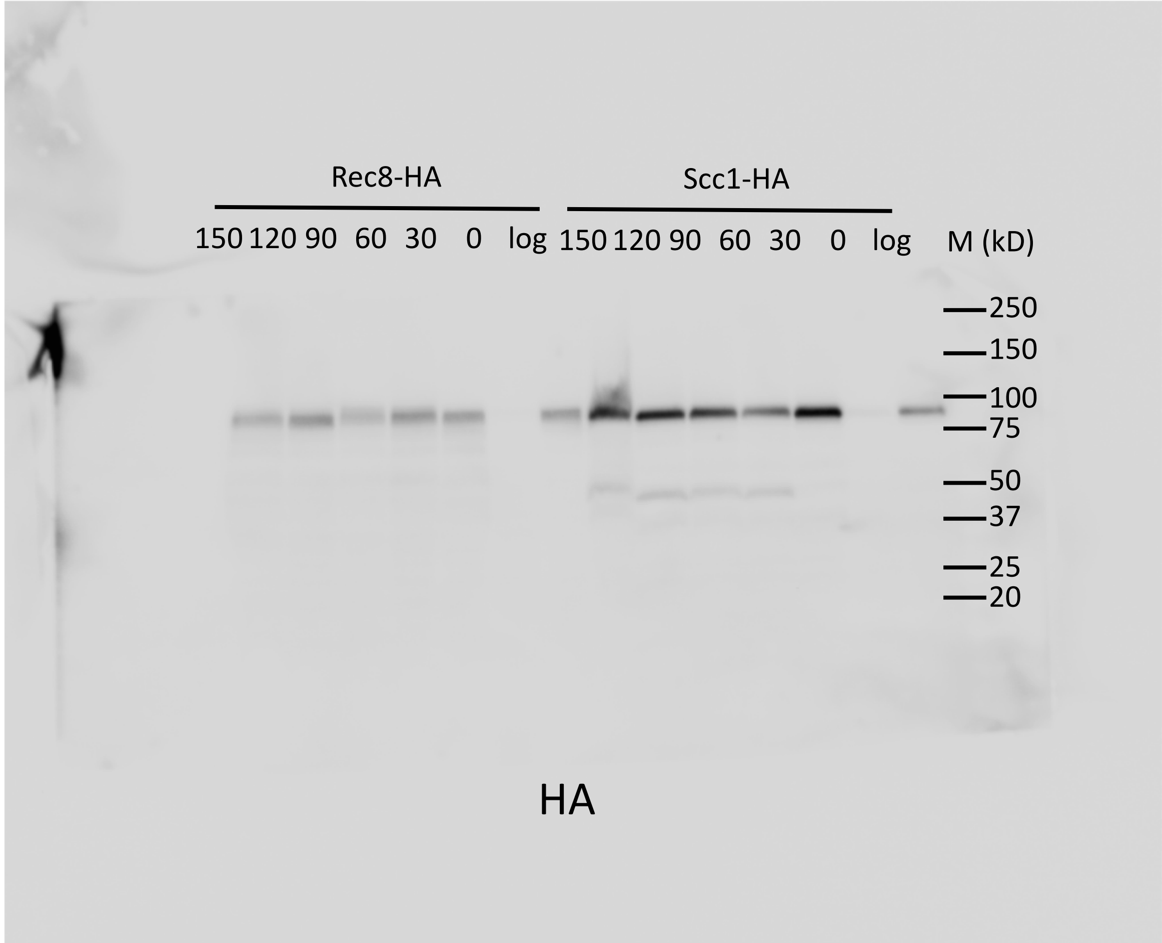

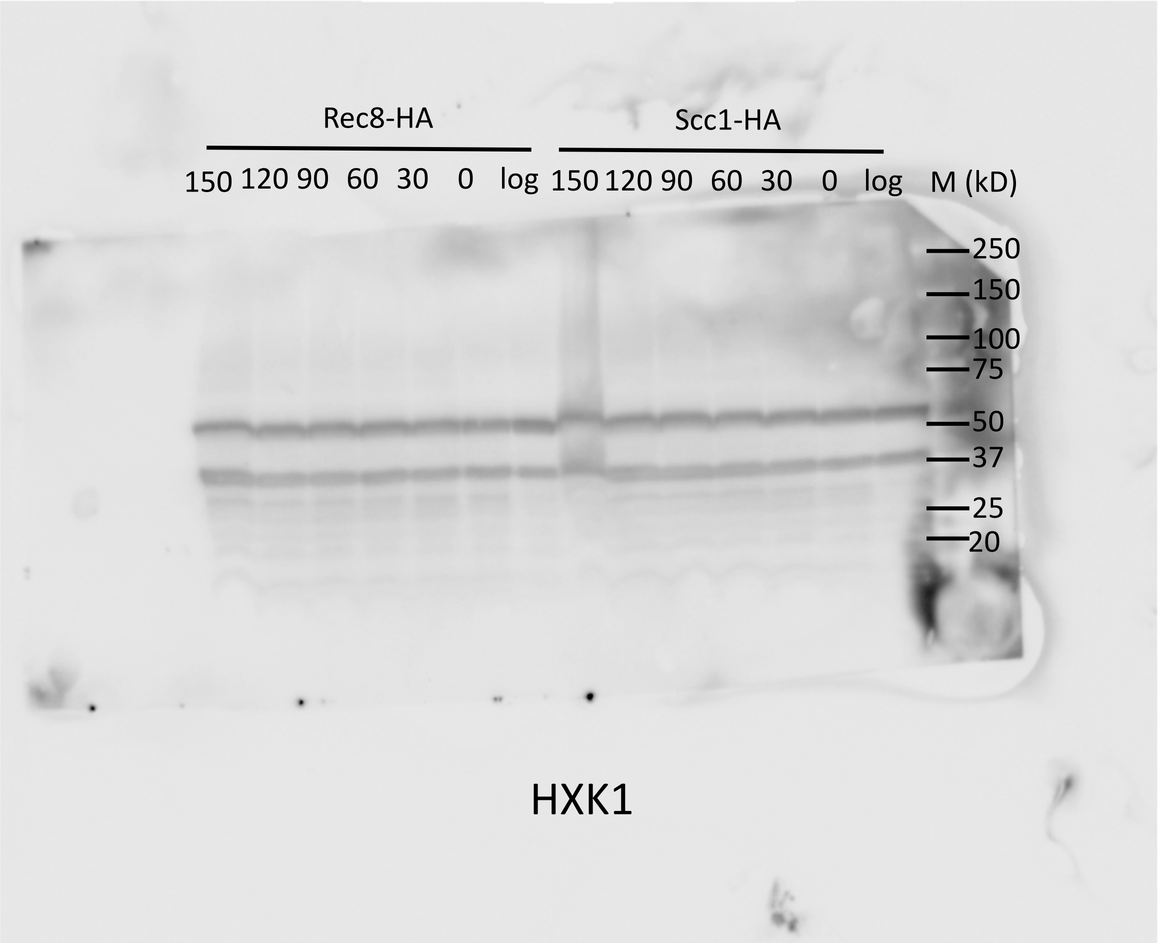

Fig 2B. Raw Image

Scc1-HA

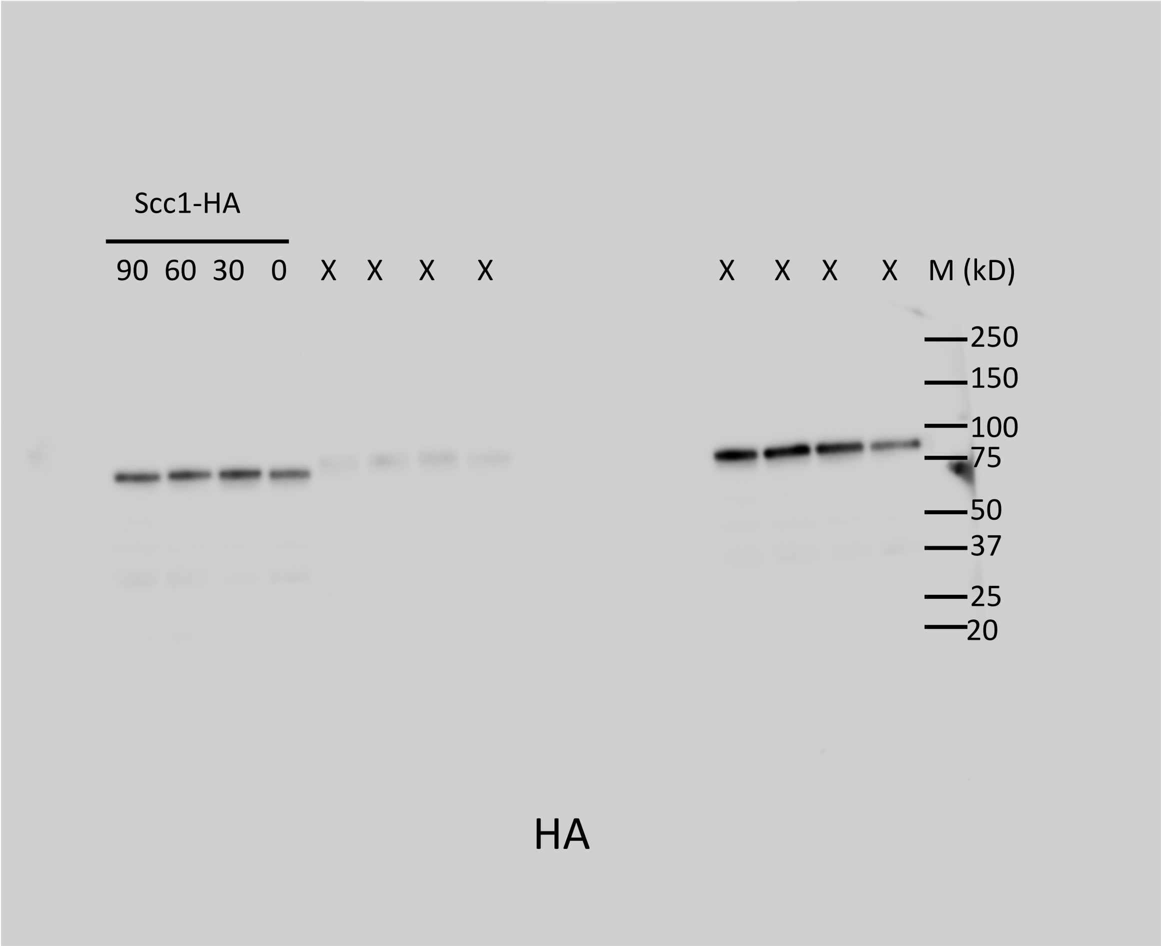

Rec8 -HA

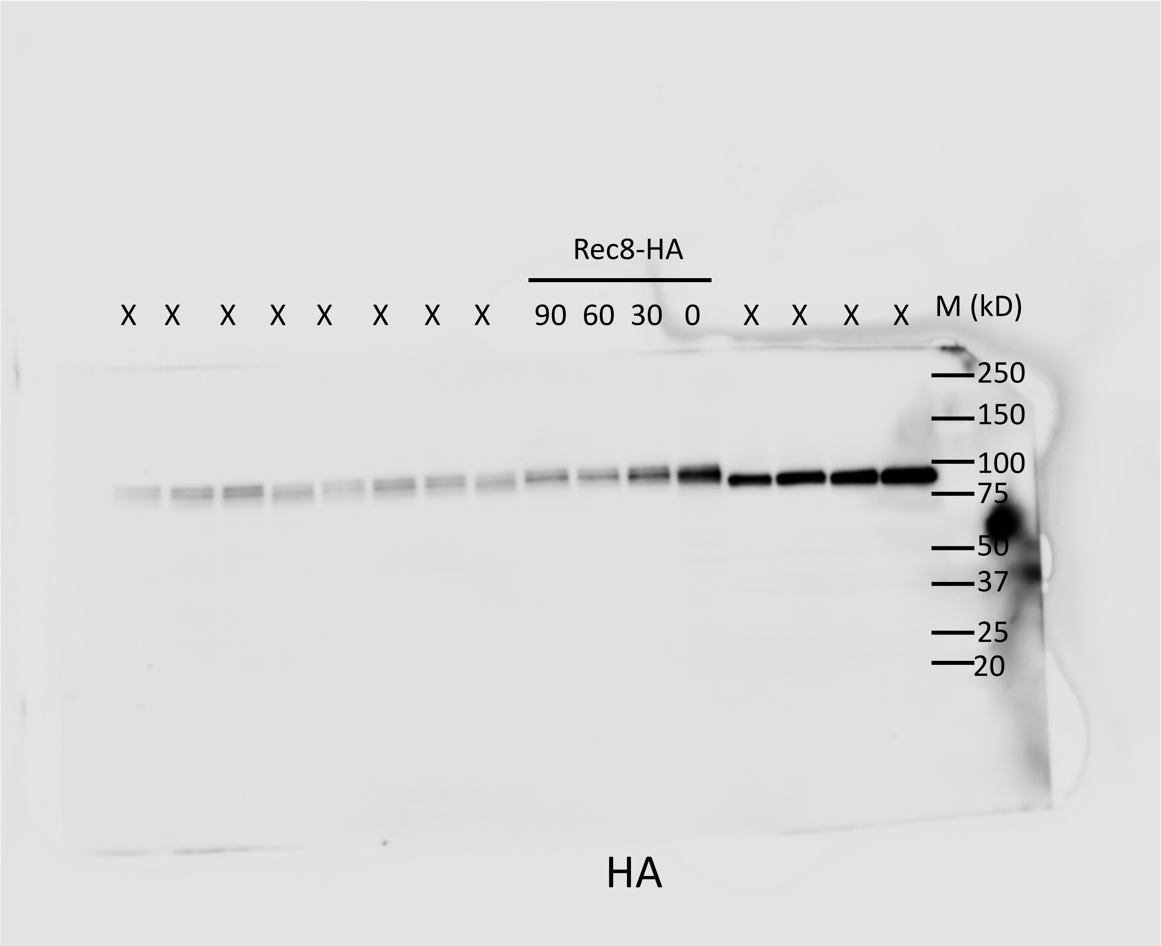

Fig 2B. Raw Image

Rec8 –HA+ *esp1-1*

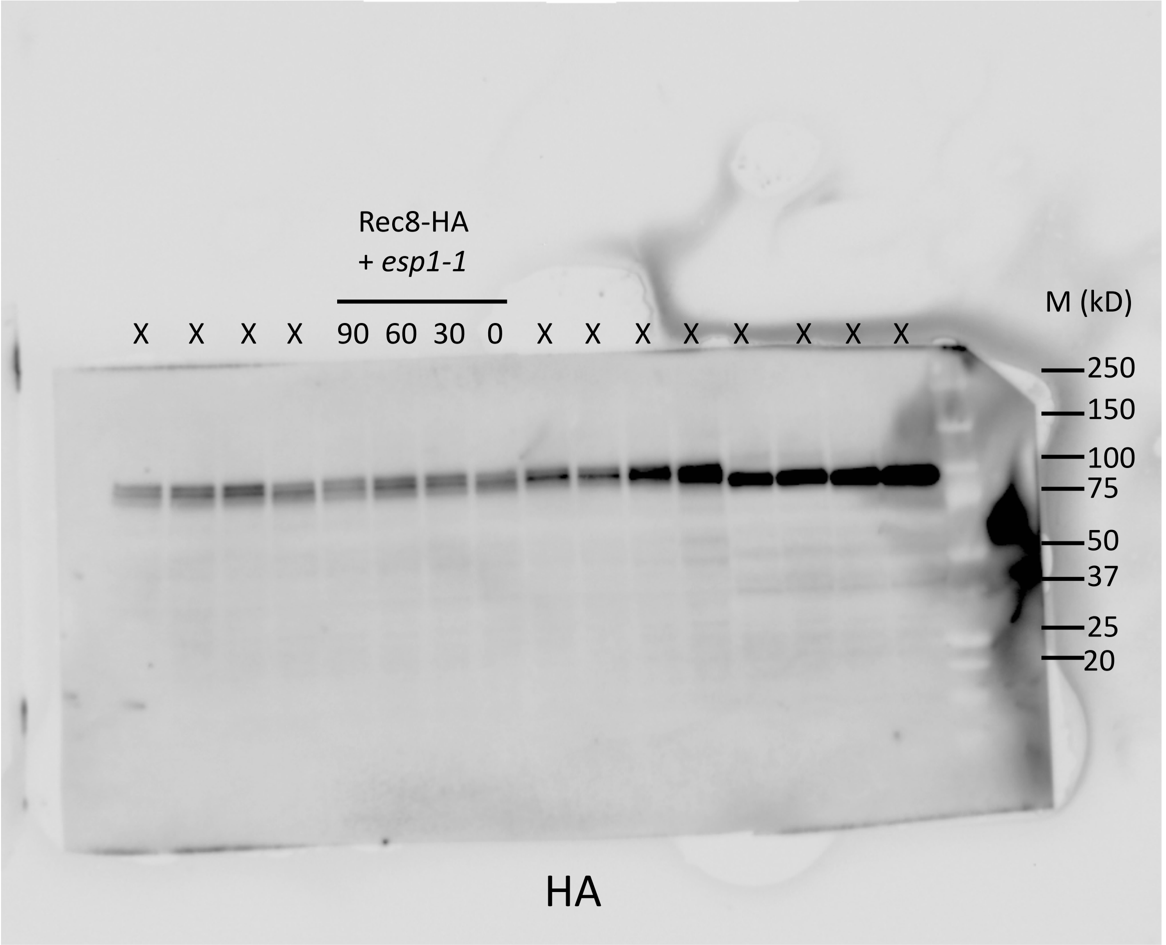

S5A Fig. Raw Image

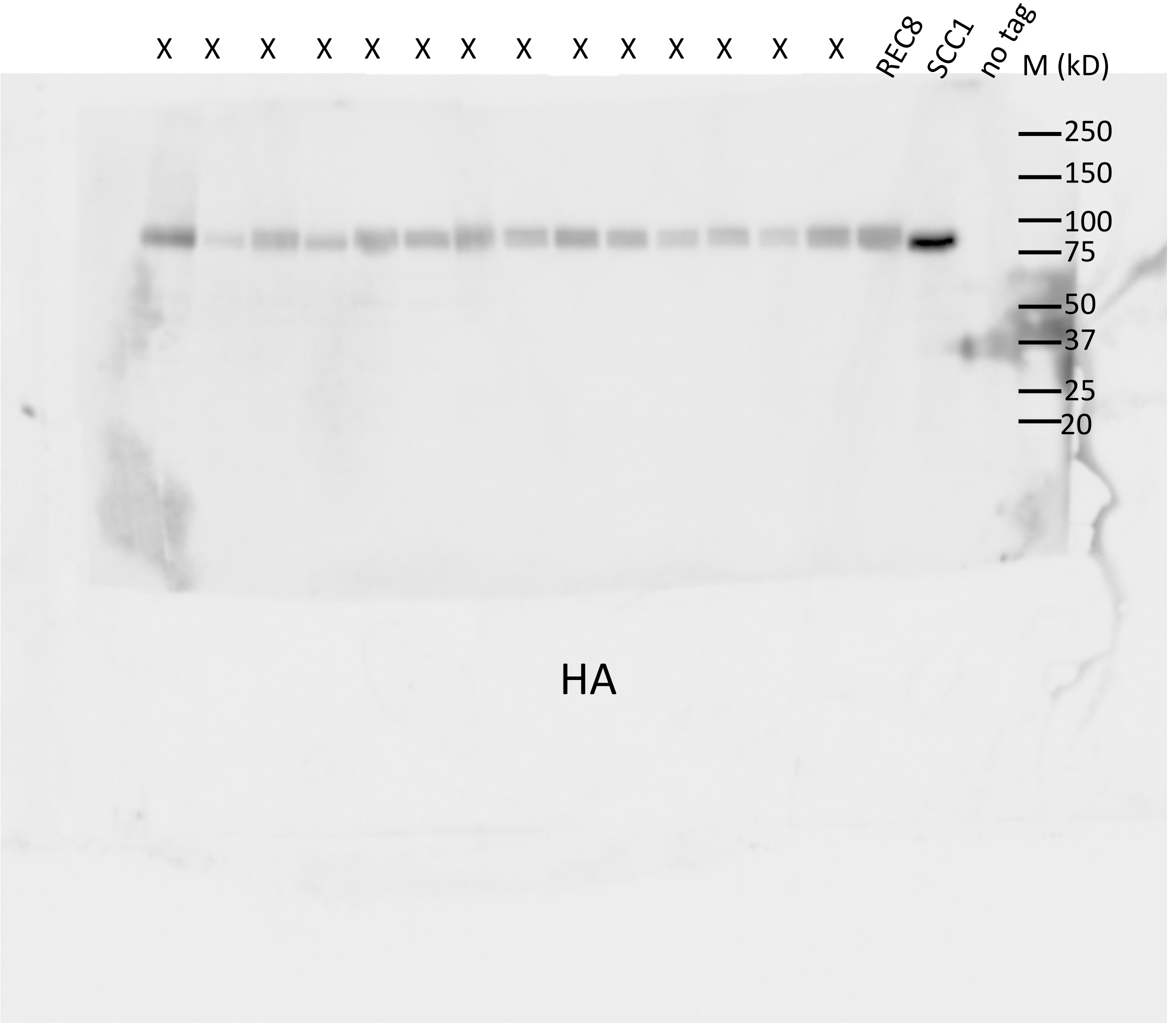

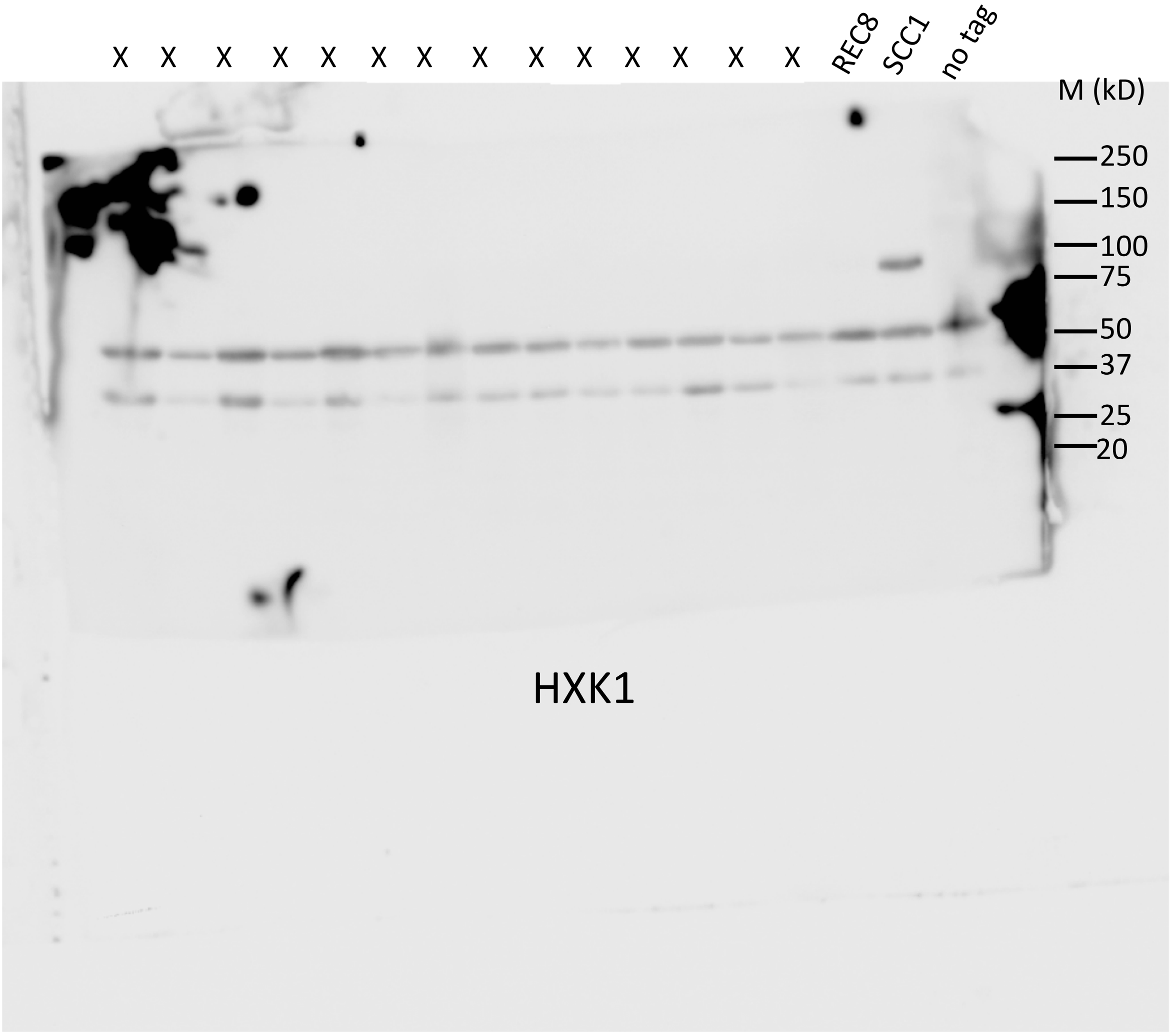

S5B Fig. Raw Image

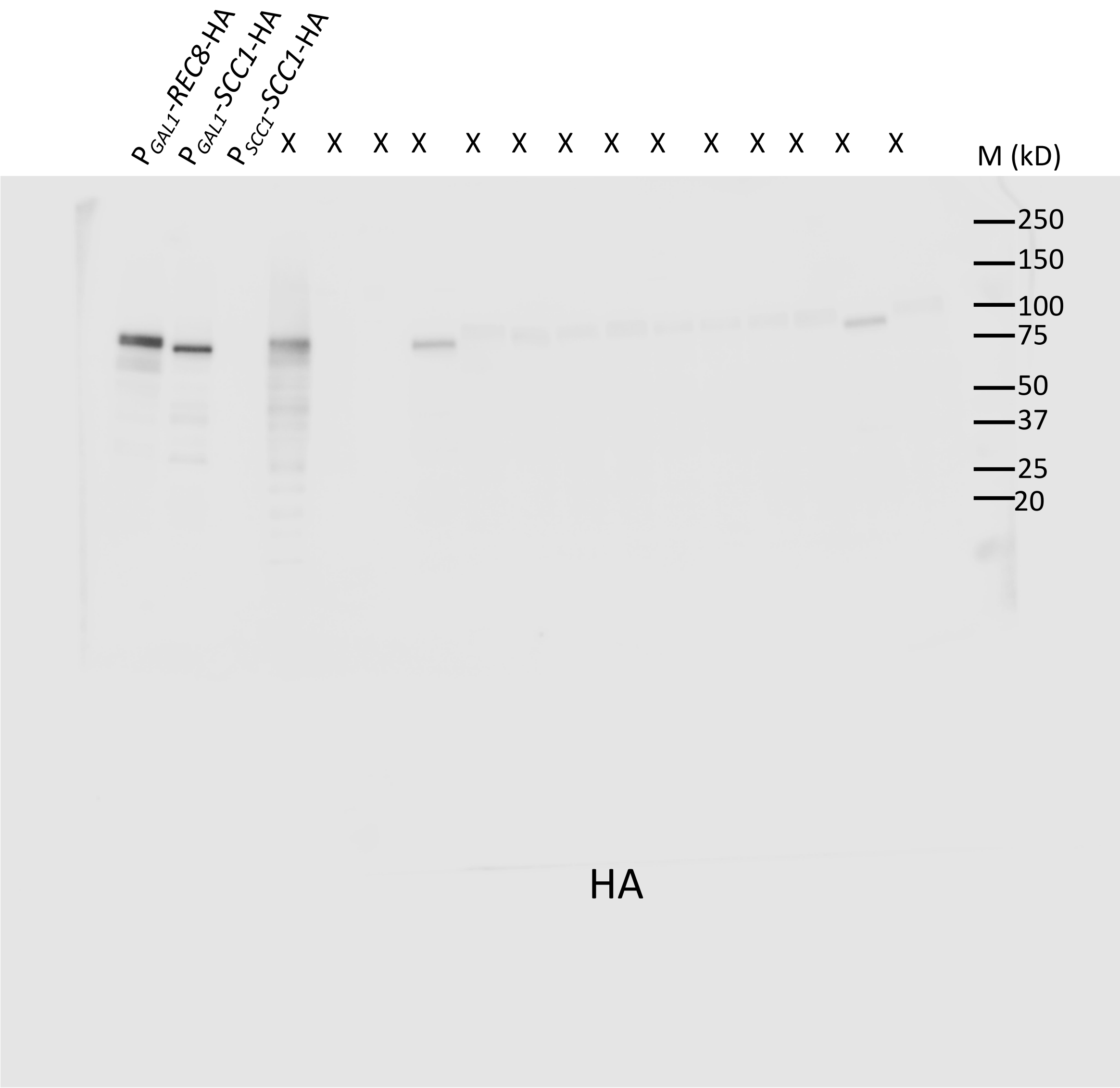

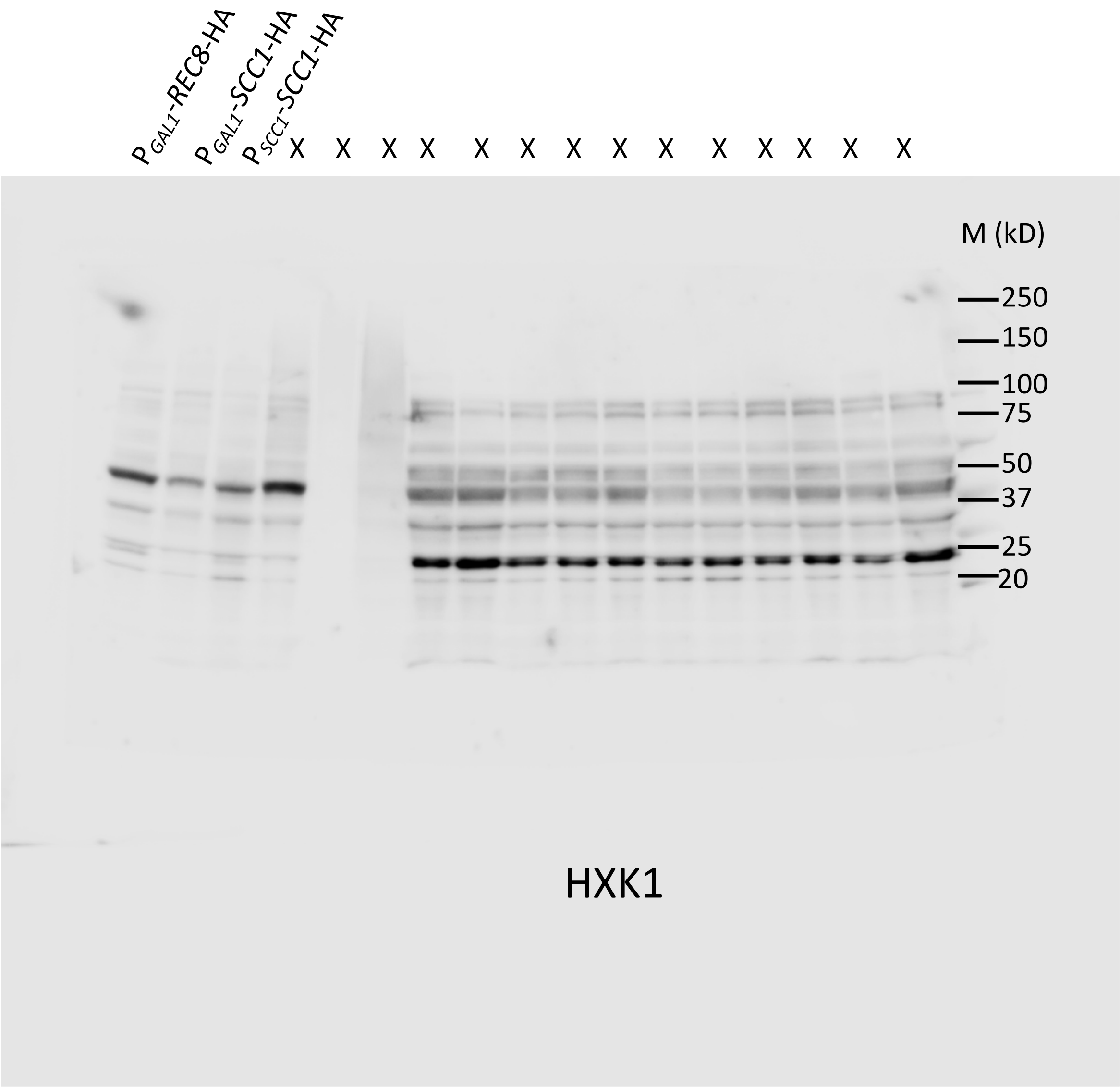

S10 Fig. Raw Image

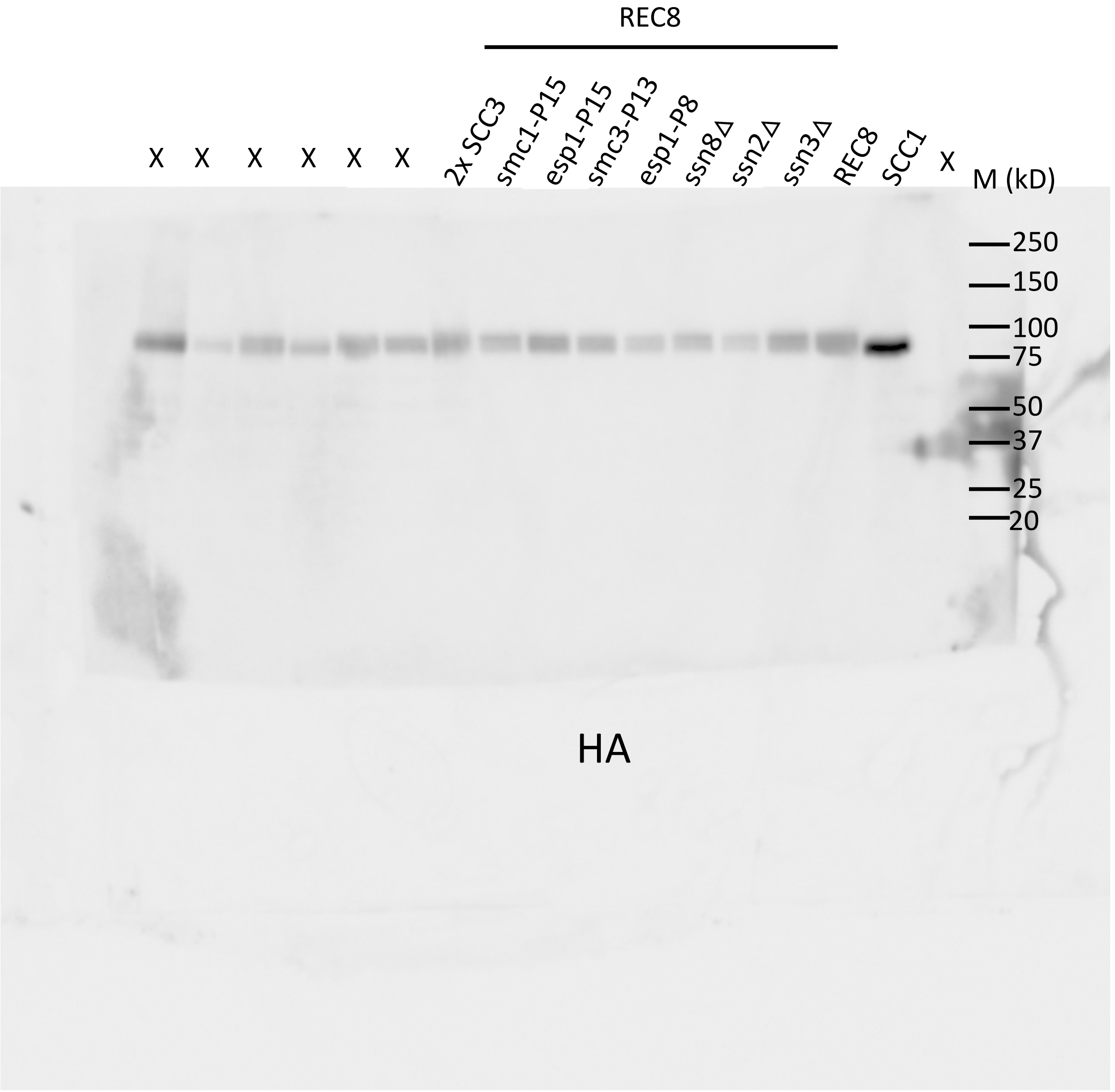

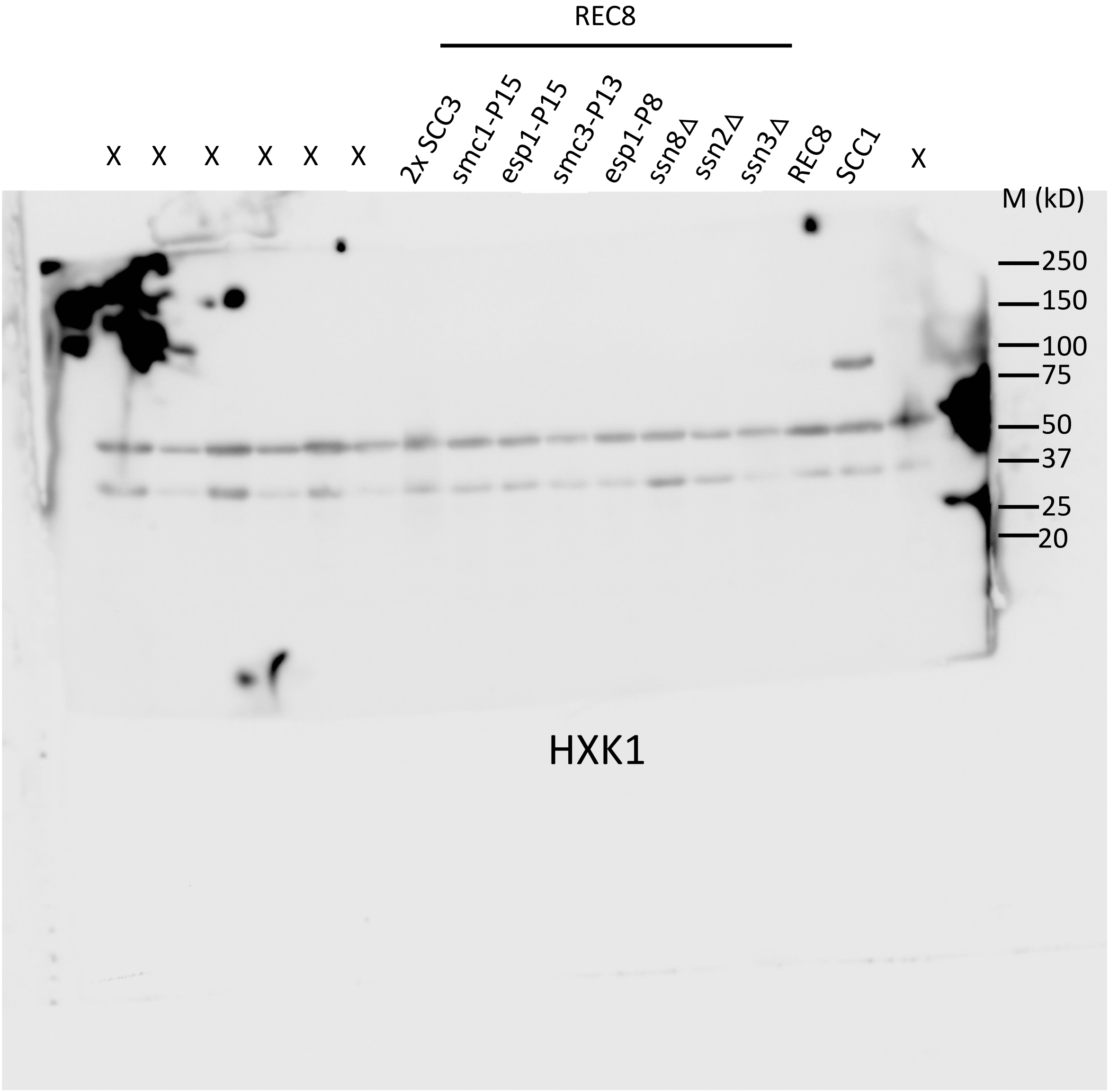

Supplement: S1 Raw Image — (PDF) [file pbio.3000635.s022.pdf]
