# Supplementary material for: Evolutionary repair: Changes in multiple functional modules allow meiotic cohesin to support mitosis
Source: PLoS Biol. 2020 Mar 10;18(3):e3000635. doi: 10.1371/journal.pbio.3000635 (PMC7138332; doi:10.1371/journal.pbio.3000635)
Supplement: Attachment — Submitted filename: Hsieh_Reply_Reviewers_Comments_final.docx [file pbio.3000635.s026.docx]

REVIEWERS' COMMENTS:

We thank editors and reviewers for reading our manuscript, giving constructive feedback, and appreciating the value of this study. We specifically address reviewers’ comments in the following comments and have revised the relevant paragraphs in the revised manuscript.

During revision, we noticed we misplaced the data of chromosomal copy of evolved clones in Fig3D. In the revised manuscript, we updated Fig3D that is plotted with sequencing data of populations and the relevant text in the result. We also updated the associated numerical values in the S1 Data.

“At generation 375, twelve populations had independently gained an extra copy of chromosome IX, and seven also had an extra copy of chromosome I or chromosome III in part of population.”

Reviewer #1:

This manuscript presents an unusual and interesting experiment. The authors test the consequences of and evolutionary response to replacing the mitotic alpha-kleisin with the diverged meiotic kleisin. First, they assay what the defects of expressing Rec8 instead of Scc1 are in their system (yeast cultures) - these include cohesion defects and earlier replication origin firing. They show that less cohesin associates with chromosomes and that cohesin is degraded faster by separase. The authors also show that the low expression of rec8 is not sufficient to account for ist lower accumulation on the chromosomes. Then they evolved 15 clones for 1750 generations and asked what the evolutionary response is to forcing cells to use REC8 in place of SCC1. They find that populations evolved mutations in several cohesin-interactors, the mediator complex, and later, mutations also in cell cycle regulators. They then functionally test these mutations and show that they do in fact improve
growth relative to the ancestral strain. They show the adaptive mutations restore sister chromatid cohesion and delay replication origin firing.

I thought this study was really interesting, thorough, and the paper well written, and I have only minor comments:

Lines 205-206: when discussing the mitotic phases, please label these on the figures (i.e. on the y axis of Fig. 1D) so the uninitiated among us know what you are referring to.

Fig.1D and its legend has been revised as reviewer 1 suggested. Because the timing of the cell cycle is different in the different strains, it’s hard to put labels on the y axis that are accurate. Instead the legend now says,

“For the cells expressing Scc1, cells are predominantly in S phase at 30 minutes, predominantly in G2 and mitosis from 45 to 75 minutes, and return to G1 at 90 minutes. Cells that express Rec8 have a long delay in mitosis, which is abolished by removing the spindle checkpoint. For examining the progression through mitosis, the timepoints from 75 to 180 minutes are highlighted by square bracket on the y-axis.”

Figure 1: Panel D - please label mitotic cell cycle phases on the y axis (or just refer to times in the text). Also - is there significance to the fact that the 180 minute peaks in scc1- and to a lesser extent in the Rec8 line are fatter and wider than those of wild type? Is replication timed more messily? Is there aneuploidy? Also, can you rule out that the bigger 2C peak is not due to polyploidy?

The *scc1Δ* cells did indeed show wider peaks than Rec8 cells at 180 minutes in our experiments. We think this widening is due to the complete lack of sister cohesion in *scc1Δ* cells. These cells eventually escape mitosis by overcoming the spindle assembly checkpoint (from 120 min to 180 min), resulting in chromosome mis-segregation that makes peaks at 180 min wider than those of WT and Rec8-expressing cells. We speculate that in Rec8-expressing cells, insufficient sister cohesion activates both spindle assembly checkpoint (due to lack of tension on sister kinetochores) and spindle orientation checkpoint (due to spindle length being constrained by residual linkage between some sister chromosomes), making escape from a mitotic arrest harder than it is in *scc1∆* cells. Please see the following cartoon.

Fig. S6 is very helpful and might be good to incorporate into Fig. 3.

We have incorporated Fig. S6 into Fig. 3

By generation 1750 several of the aneuploids for Chr 9 had lost the extra chromosome. Did they gain a local duplication or change in expression of SCC3, or did the additional mutations that evolved obviate the extra SCC3?

We did not find any mutations in the promoter and coding sequence of *SCC3* in the populations that lost the extra copy chromosome IX. In our genome sequencing data, the copy number of *SCC3* was consistent with there only being a single copy of the gene. We also did not find a strong correlation between adaptive mutations we identified and the loss of the extra copy chromosome IX. We speculate that the loss of the extra copy of chromosome IX in a fraction of our populations reflects different balances between the benefit of an extra copy of *SCC3* compared to the cost of unbalanced gene expression. We added the following text to the discussion.

“We demonstrated that gaining an extra copy of chromosome IX, increasing the copy number of *SCC3*, could be beneficial to Rec8-expressing cells; however, at generation 1750, five out of twelve populations lost one extra copy of chromosome IX, suggesting the benefit of chromosome IX disome is transient in some evolved populations. We did not find any mutation or increased copy number of *SCC3* in these five populations, suggesting the loss of chromosome IX disome is not associated with the alteration of *SCC3*. We speculate that the fitness effect of gaining an extra copy of *SCC3* competes with the cost of unbalanced gene cause by an extra copy of chromosome IX and the balance between the cost and benefit changes during the evolutionary trajectories of our populations. Because the segmental duplications were rare, we cannot conclude that they are beneficial. The segmental duplication on chromosome V could promote adaptation of Rec8-expressing cells because the duplicated region contains *SCC4*, one component of the cohesin loading complex. Duplication of this gene improves sister chromosome cohesin in yeast cells adapted to replication stress [36].”

Line 40: «direct and direct» - you probably mean direct and indirect?
Line 47: Perhaps «answering this question» instead of «this problems»
Line 245: «In the bar graph, the upper arrow marks…» - I think you mean in the blot.

Thank you. We have corrected these errors.


Reviewer #2:
[identifies himself as Andreas Hochwagen]

This study investigates the evolutionary cost associated with the sub-functionalization of the cohesin complex in budding yeast. Mitotic and meiotic yeast use cohesin complexes that differ in their kleisin subunits. Previous work had found that expression of the meiotic kleisin, REC8, in place of mitotic kleisin, SCC1, leads to fitness defects. The authors now show that this defect is linked to incomplete sister chromatid cohesion, leading to increased rates of chromosome misalignment and premature loss of cohesion.  They also observe an unexpected acceleration of DNA replication timing in mitotic cells expressing REC8. Evolution experiments identified a number of spontaneous suppressor mutations of these defects. Several of the suppressor mutations affect the mediator complex, the cell cycle entry machinery or cohesin regulation, but no mutations were observed in REC8. Intriguingly, all of these suppressors appear to rescue the early S phase phenotype of REC8 misexpression.

This is a very nice and interesting paper. The writing is clear and the experiments are generally conclusive. I only have a few minor comments.

1.      I wonder whether the accelerated S phase of REC8-expressing cells could be related to defects in recruiting Rad53. The Longhese group showed a number of years ago that meiotic chromosomes do not activate Rad53. Since Rad53 is an important factor in regulating the timing of origin firing, a failure to properly activate Rad53 may lead to accelerated S phase. Exploring this possibility is obviously beyond the scope of this paper, but it may be worth considering this possibility in the discussion.

We thank reviewer 2 for proposing this interesting hypothesis and we mention it in the discussion:

“Our work leads to a number of questions. Why does expressing Rec8 advance the timing of origin firing while removing Scc1 has no effect? How do mutations in transcriptional mediator and cohesin-related genes affect replication? Are the effects of Rec8 on replication in the mitotic cycle related to its reported ability to stimulate genome replication in the meiotic cycle [66]? One possibility is that Rec8 affect replication by altering the level of chromosome-bound Rad53, a checkpoint protein that can regulate origin firing [67] and whose role has been reported to be altered in meiosis [68].”

2.      One thing that needs clarification is the mutation calling for REC8. The authors speculate that the absence of mutations in REC8 reflects differences in target size between the observed suppressors and REC8. However, the way the experimental strain is constructed, REC8 is present in two copies: one on the plasmid under the SCC1 promoter, and one in the genome under its own promoter. As a result there will always be a 50% background from the unexpressed genomic REC8 copy in the sequencing reads. The authors focus only on fixed mutations, and define fixed mutations as mutations found in >90% of reads. This cutoff would make it impossible for rec8 mutations to reach fixation given the expected 50% wild-type sequences from the genomic REC8 copy. This should be clarified and probably warrants reanalysis of the REC8 sequences with a different cutoff.

We thank reviewer 2 for this insight. We searched for all mutations that were present at >10% in the evolved populations and added this data as supporting file 3. Using the 10% mutation cut off, we did not find any Rec8 mutation in any population at generation 375. But we did find one Rec8 mutation (E148Q) present in population P12 at generation 1750 (at the frequency of 85%). This mutated residue is not located in the domains that interact with Smc proteins or other known functional motifs, and since it occurred in only one population we cannot reliably infer that the mutation confers a fitness effect. We now mention and discuss this one population in the text.

*Results:*

“We also searched for mutations in *REC8*, either in the coding sequence or the DNA 500 bp upstream and downstream of the ORF, using a lower threshold because our strains have two copies of *REC8*, one on the plasmid, driven by the *SCC1* promoter, and one on the chromosome driven by the *REC8* promoter. Population 12 had a single mutation (E148Q) on the plasmid-borne copy of *REC8*. Because this substitution is a conservative amino acid change, the position lies outside the regions of kleisin that are known to have interactions with other protiens and it occurred in only one population we do not know if it is adaptive or a chance event.”

*Discussion:*

“We argue that considering the target size for different mutations explains why we saw only one mutation in Rec8. Since Rec8 and Scc1 have diverged substantially roughly a billion years, it may require multiple, simultaneous amino acid substitutions in Rec8 to improve its ability to hold mitotic sister chromosomes together.”

3.      Were any partial aneuploidies observed or were all aneuploidies affecting entire chromosomes?

At generation 1750, we detected two segmental duplication events. One of the segmental duplications is on chromosome V. This duplication was found in another evolution experiment in the Murray lab, where it contributes to the fitness of cells lacking the replication protein, Ctf4 (which also has a role in sister cohesion) by producing an extra copy of *SCC4*, which encodes one subunit of the cohesin loader. The other duplication is on chromosome IV, spans 112 kb, and lacks any obvious candidate gene to confer a fitness advantage. Since segmental duplications are rare in this experiment, we are not sure if these two events were selected. We have added the following sentences to the results and discussion.

*Results:*

“We also detected two segmental duplication events at generation 1750: population P4 duplicated part of chromosome IV and population P7 duplicated part of chromosome V (Fig. S6B and C). These duplicated regions are flanked by Ty1 transposons, suggesting segmental duplication is a result of recombination between homologous transposon sequences. The duplication on chromosome V contains *SCC4*, whose duplication increases the fitness of strains that have cohesin defects that result from removing Ctf4 [36] and the other duplication lacks obvious candidate genes making it unclear whether it was selected or is a chance event.”

*Discussion:*
“We see frequent aneuploidy for chromosome IX and two different examples of segmental aneuploidy, each occuring in only one population. The prevalence of aneuploidy compared to segmental duplication in our evolution experiment is likely to be explained by frequent chromosome mis-segregation in Rec8-expressing cells. We demonstrated that gaining an extra copy of chromosome IX, increasing the copy number of *SCC3*, could be beneficial to Rec8-expressing cells; however, at generation 1750, five out of twelve populations lost one extra copy of chromosome IX, suggesting the benefit of chromosome IX disome is transient in some evolved populations. We did not find any mutation or increased copy number of *SCC3* in these five populations, suggesting the loss of chromosome IX disome is not associated with the alteration of *SCC3*. We speculate that the fitness effect of gaining an extra copy of *SCC3* competes with the cost of unbalanced gene cause by an extra copy of chromosome IX and the balance between the cost and benefit changes during the evolutionary trajectories of our populations. Because the segmental duplications were rare, we cannot conclude that they are beneficial. The segmental duplication on chromosome V could promote adaptation of Rec8-expressing cells because the duplicated region contains *SCC4*, one component of the cohesin loading complex. Duplication of this gene improves sister chromosome cohesin in yeast cells adapted to replication stress [36].”

4.      Line 537: I am not sure I understand the argument put forward about the relative origin firing. I agree that earlier origins generally have a lesser chance of being inactivated by nearby origins, but the authors also show that the temporal order of origin firing does not seem to be affected. Doesn't that mean that speeding up replication for any particular origin is irrelevant because all the neighboring origins will also speed up proportionally? At least based on this I would assume that there is no increased chance of avoiding inactivation by a neighboring origin.

Thank you. The reviewer is entirely correct. Our writing and thinking was sloppy and we have removed the offending sentence.

Typos:
Line 122: Scc1-expressing
Line 328: both CLN2 and SWI4 mutated
Line 564: which delays replication origin firing

Thank you. We have corrected these errors.


Reviewer #3:

Hsieh and colleagues use experimental evolution to examine if and how cells affected in mitotic cohesion function are able to restore fitness.  They find that replacing the mitotic kleisin gene SCC1 with its meiosis-specific paralog REC8 results in a fitness decrease due to reduced cohesin activity.  Evolving 15 parallel mutant populations for 1750 generations partly restores fitness through a combination of mutations located in in loci related to the mediator complex (Cdk8 module), cohesin function (ESP1, SMC1 and SMC3) and cell cycle regulation (MBP1, CLN2).  Interestingly, none of the compensatory mutations were located in or near the REC8 gene, suggesting that the fitness effect of single mutations in REC8 is likely limited, while the frequency of combinations of mutations in REC8 that may confer stronger fitness benefits, is too low.

This is an exceptionally eloquent paper that draws from an impressive body of work. Perhaps the biggest concern is that the study does not bring much truly novel biological insight.  That said, the paper does yield two major conclusions.  Firstly, it helps to shed light on the biological processes controlling cohesin activity.  Specifically, the results show how proper cohesin function does not only require proper assembly of the cohesion complex, but also a sufficiently slow progression through S phase as well as some -as yet unknown- mediator activity (nicely summarized in Fig 8).  Second, the results remind us how evolving suppressor phenotypes often depends on mutations that occur relatively frequently, such as structural variation and (partial) loss-of-function mutations.  This may be especially the case in laboratory conditions, where such relatively "blunt" evolutionary solutions may not have the same pleiotropic (negative) fitness consequences as they would have in
more complex natural settings.  Perhaps this second point should be stressed even more in the discussion section.

Minor remarks

I found Figure 1 panel A to be a bit cluttered and unclear; pls consider refining

Thank you. We have revised Fig1A to reduce clutter

.
Figure 3B - would it perhaps make more sense to compare everything to the WT fitness level?  Also, pity that the WT was not evolved in parallel with the REC8 mutant.

Experimentally, fitness measurements are most accurate when the difference in the fitness of the two strains is modest. Since we didn’t know how much the fitness would increase, we choose to measure fitness against the ancestral, *P_SCC1_-REC8* strain rather than the wild-type, *P_SCC1_-SCC1* strain. Once we had picked a comparison strain, we needed to stick with it and thus we measured all fitness (including the wild-type strain, evolved strains, and reconstructed strains) against a fluorescently-labeled *P_SCC1_-REC8*.

There have been a large number of experiments that have evolved W303 and S288c backgrounds to grow better on the medium (YPD) we used for our experiment. In particular, another paper from the Murray lab [36] did the precise control of evolving WT in parallel. None of our adaptive mutations appear in this wild-type evolution experiment strongly suggesting that the mutations that we identify as adaptive are specific to Rec8-expressing cells. Both the original and the revised text include this statement:

“The genetic alterations we found are specific to yeast cells adapting to expressing Rec8 rather than Scc1. Mutations in transcriptional mediator, cohesin-related genes, and the G1-to-S regulators have not been seen at frequencies that suggest they are adaptive in previous experimental evolution studies in *S. cerevisiae* [36-40]”

The text is a bit long and therefore loses a bit of its poise and punch.

Our goal was to try and make the paper as accessible as possible and there’s always some compromise between explaining things clearly and keeping the paper short.


REQUEST FROM THE ACADEMIC EDITOR:

[I would like to see] a more nuanced discussion of paralog divergence (e.g., what would the ancestral situation have been? what are the effects of the adaptive mutations in the context of normal Scc1 or in the context of Rec8 functioning during meiosis?).

Thank you. We now discuss paralog evolution in the discussion and raise both of the questions that the academic editor mentions.

“Our results do not distinguish between two possible trajectories that led to the functional separation between the two current kleisin paralogs: i) a single kleisin cannot easily support the different forms of chromosome segregation seen in mitosis and meiosis and this incompatibility forced the two kleisin paralogs to diverge from each other, and ii) mutations that impaired Rec8’s ability to support mitosis accumulated by genetic drift rather than selection. Our work reveals the power of a variety of adaptive mutations, affecting diverse modules, to alter the ability of a protein to participate in a biological function. How specific are the benefits of the mutations that we observed to cells using the meiotic kleisin in the mitotic cell cycle? This question could be answered by investigating their effects in two contexts in wild-type cells: in mitotic cell cycles that used the mitotic kleisin, Scc1, and in meiosis, with Rec8 expressed from its normal promoter”
